# Apoptosis promotes fertility in *Caenorhabditis elegans* by maintaining functional germline morphology

**DOI:** 10.1242/dev.205442

**Published:** 2026-08-13

**Authors:** Udodirim N. Saydee-Onwubiko, Michael E. Werner, Gavin C. Trapp, Amy S. Maddox

**Affiliations:** Department of Biology, University of North Carolina, Chapel Hill, NC 27599, USA

**Keywords:** Apoptosis, Oogenesis, Germline architecture, Infertility, *C*. *elegans*

## Abstract

Programmed cell death (apoptosis) during oogenesis is conserved across metazoans and linked to regulation of oocyte number and quality. In oogenic germlines, the removal of developing oocytes by apoptosis ensures that oocytes do not contain DNA damage or multiple nuclei. Beyond this chromatin-quality control assurance role, it was unknown how apoptosis contributes to oocyte quality. We used the nematode *Caenorhabditis elegans* to study the consequences of loss of apoptosis on oogenesis. Blocking apoptosis reduced fecundity in hermaphrodites at peak fertility and caused germline architectural defects, such as abnormal rachis morphology and perturbed arrangement and distribution of oogenic germline compartments. Our results suggest that the loss of germline apoptosis arises due to lack of sufficient space for, and reduced cytoplasmic flows into, developing oogonia. In support of this idea, oocytes and embryos are abnormally small and exhibit low viability in animals unable to execute apoptosis. These findings suggest that, in addition to preventing ploidy defects during oogenesis, apoptosis contributes to fertility by preserving the homeostatic germline structure required for the fidelity of oogenesis.

## Teaser

**Highlighted Article:** Blocking apoptosis during *C. elegans* oogenesis disrupts germline structure over time, causing morphological defects and infertility, highlighting the role of apoptosis in maintaining syncytial architecture and reproductive function.

## INTRODUCTION

The US National Institutes of Health reported that one in four women of reproducing age experience infertility directly linked to oocyte quality defects, highlighting a need to understand the cellular mechanisms that ensure the fidelity of oocyte production or oogenesis (Nugent and Chandra, 2024; Pfennig et al., 2022). Oocyte quality (ploidy, size and general physiology) is directly proportional to embryo fitness and viability (Conti and Franciosi, 2018; Hughes et al., 2018; Maddox et al., 2005). Cell size is of particular importance for oocyte function, since the egg must contain all the biomass necessary for development until the embryo can access outside nutrients. The mechanisms that ensure oocyte quality is achieved and regulated are poorly understood (Frost et al., 2023; Luo et al., 2010).

Germ cell cysts in insects and vertebrates, as well as oogenic germlines in nematodes, are syncytia (Hubbard and Greenstein, 2005; Spradling, 2024). Within these syncytial structures, germ ‘cells’ are compartments in which germline nuclei reside, interconnected by actomyosin-rimmed cytoplasmic bridges (Edwards and Kiehart, 1996; Gerhold et al., 2022; Pepling and Spradling, 2001). In the germline, oogenic germ cells undergo mitotic divisions, become meiotic, and eventually experience one of two fates: elimination by apoptosis, or maturation into oocytes (Pepling, 2006; Pepling and Spradling, 2001). In all organisms examined to date, apoptosis during oogenesis occurs in germline compartments that are in the pachytene stage of meiosis I prophase (Du Pasquier et al., 2011; Kim et al., 2013; Niu and Spradling, 2022). Generally, apoptosis is essential for shaping tissues during development, and eliminating defective cells, which ensures homeostatic health and the avoidance of pathologies, including cancer (Lindsten et al., 2000). During oogenesis in humans, mice and *Drosophila*, apoptosis removes >90% of developing oocytes (Tilly, 2001). Mutations in apoptosis-related genes have been linked to maternal fertility defects in mammals (Ratts et al., 1995; Veis et al., 1993). In some species, apoptotic compartments serve a nurse cell function, providing cytoplasm to promote oocyte growth and development (McCall and Steller, 1998; Technau et al., 2003). In *Drosophila*, elimination of 15 of 16 oogonia by apoptosis is essential for oocyte enlargement and therefore viability (McCall and Steller, 1998). By contrast, in mammals, most growth of oocytes occurs after the breakdown of the germ cell cyst when apoptosis is prevalent (Anderson and Telfer, 2018; Niu and Spradling, 2022). In nematodes, cytoplasm from apoptotic compartments is thought to contribute a small amount of the massive volume of cytoplasm taken up by nascent oocytes prior to cellularization (Wolke et al., 2007); apoptosis has primarily been linked to ensuring elimination of compartments containing multiple nuclei or DNA damage (Raiders et al., 2018).

**Fig. 1.**
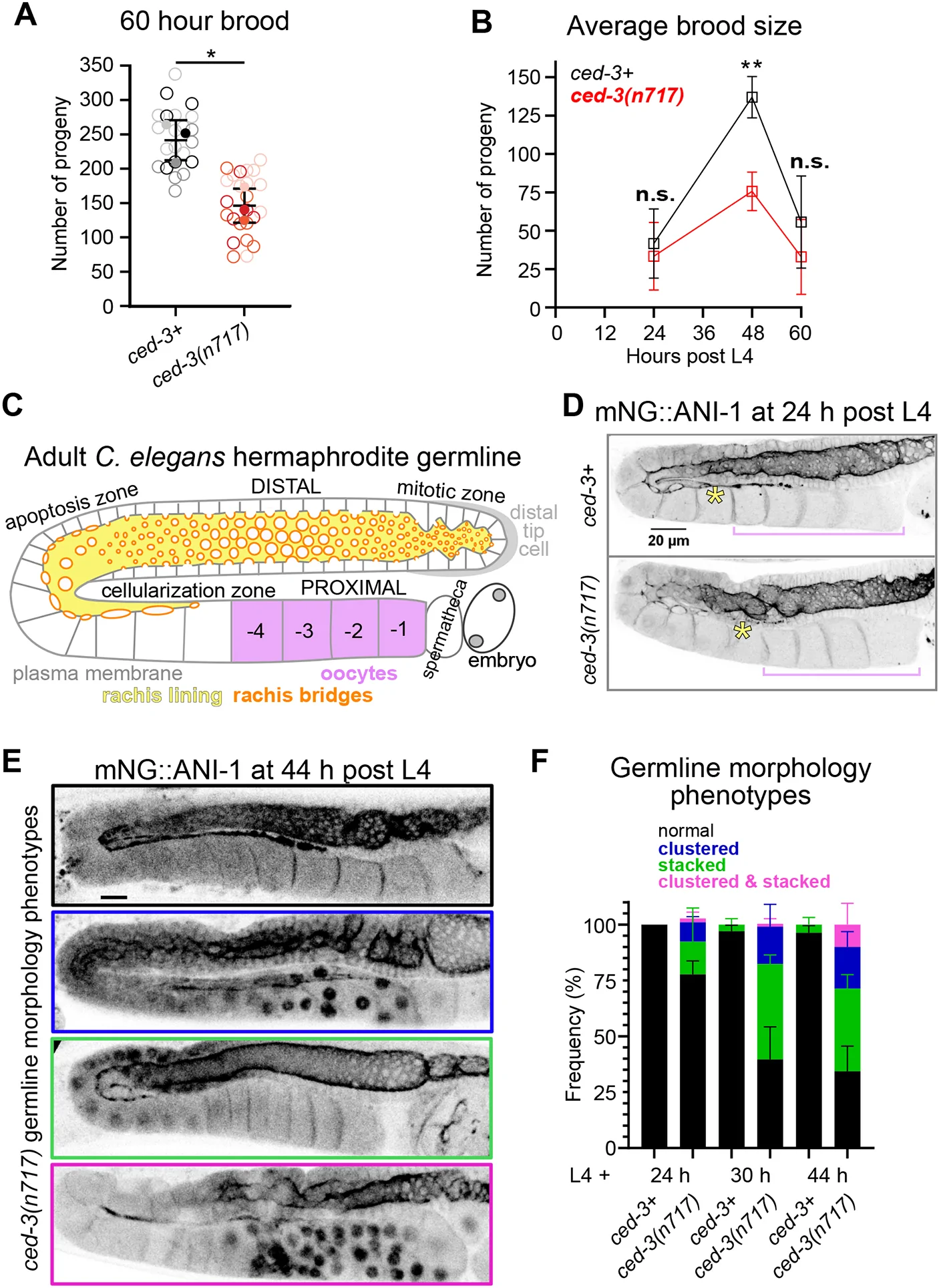
***ced-3(lf)* hermaphrodites exhibit brood size and germline architecture defects that intensify over the reproductive lifespan.** (A) Quantification of total brood size in the indicated genotypes. Data points represent individual worms from three independent experiments (various gray levels) (*n*≥10 hermaphrodites per condition analyzed). *P=0.013 (Welch's two-tailed *t*-test). (B) Quantification of average brood size in time windows 0-24, 24-44 and 44-60 h post L4. Data points represent individual worms from three independent experiments (*n*=24 animals per genotype). n.s.: 24 h, *P*=0.671; 60 h, *P*=0.370; **P=0.0046 (ANOVA followed by Šídák's post-hoc analysis). (C) Schematic of an adult *C*. *elegans* hermaphrodite germline highlighting key morphological features and zones. (D) Images of control and *ced-3*(*lf*) germlines at 24 h post L4 from animals expressing mNG::ANI-1 from its endogenous locus (MDX29 and MDX129). Asterisks indicate the point of rachis closure (cellularization), and magenta brackets indicate oocytes. (E) Representative images of germlines in animals expressing mNG::ANI-1 showing the germline morphologies scored in F: black, normal; blue, clustered germ compartments; green, stacked oocytes; magenta, clustered germ compartments+stacked oocytes. (F) Quantified frequency of germline morphologies of control and *ced-3*(*lf*) animals at different reproductive timepoints (L4+24 h, L4+30 h and L4+44 h; three independent experiments, *n*≥80 animals per experimental condition). Error bars represent mean±s.d. Scale bars: 20 μm.

Apoptosis is initiated by the activation of proteases called caspases that degrade proteins to dismantle cellular structures (Kumar, 2007). The presence of multiple caspase pathways in most metazoans confounds deciphering the roles of apoptosis regulators in oogenesis (Khammari et al., 2011; McCall, 2004; Perez et al., 2007; Veis et al., 1993). In *Caenorhabditis elegans*, the effector caspase CED-3 is the sole caspase necessary and sufficient for germline apoptosis; loss of CED-3 is an elegantly simple way to experimentally tune apoptosis in this animal (Gumienny et al., 1999). The stereotypic and well-characterized development and function of the hermaphrodite germline also facilitate interrogation of the roles of apoptosis in *C. elegans*. Hermaphrodites first generate ∼300 sperm, and transition into oogenesis in the fourth and final larval stage (L4) (Lints and Hall, 2009; Pazdernik and Schedl, 2013). At 24 h post L4, animals are young adults, and their germlines make oocytes that are fertilized by sperm stored in the spermatheca (Pazdernik and Schedl, 2013). The oogenic germline consists of two U-shaped tubes that each connect, via a spermatheca, to a central uterus (Kimura and Motegi, 2025). In a stem cell niche in the distal tip of the germline, germline stem cells undergo mitotic proliferation (see Fig. 1C). When they are displaced out of the niche, germline compartments transition into meiosis. Approximately 50% of compartments in pachytene of meiosis I prophase undergo apoptosis (Gumienny et al., 1999), while the rest exit pachytene in a Ras/MAP kinase-dependent manner, progressing through diplotene and diakinesis until they receive signals to complete maturation and be ovulated (Church et al., 1995; Huelgas-Morales and Greenstein, 2018; Lee et al., 2007b). Once ovulated from the proximal germline into the spermatheca, oocytes are fertilized, and, once in the uterus, complete meiosis and begin embryogenesis (Hubbard and Greenstein, 2005). *C. elegans* hermaphrodites' period of fertility depends on the presence of sperm, which is finite in the absence of mating; exhaustion of the sperm supply marks post-fertility (McCarter et al., 1999; Scharf et al., 2021).

The *C. elegans* caspase CED-3 is activated through physical interaction with the pro-apoptotic Apaf-1 homolog CED-4 (Hengartner et al., 1992; Wu et al., 1997). Mutations in *ced-3* or *ced-4* that alter their physical interaction phenocopy each other (Gumienny et al., 1999; Hengartner et al., 1992). How CED-3-driven apoptosis affects oocyte quality is not understood. Aged, post-fertility *ced-3*(*lf*) mutants have been reported to exhibit germline hyperplasia (Gumienny et al., 1999) and reduced oocyte quality (Andux and Ellis, 2008). However, other work reports that *ced-3*(*lf*) animals are viable, fertile and have normal germline architecture when actively producing oocytes that are fertilized (peak fertility) (Das et al., 2020). To address this conundrum and understand the cellular implications of apoptosis in oogenesis, we tested whether blocking apoptosis perturbs germline morphology and reproductive success, across several developmental timepoints. We discovered that blocking apoptosis reduced maternal fertility much earlier than previously reported (Andux and Ellis, 2008; Gumienny et al., 1999), within the timeframe of normal fertility. Apoptosis-defective germlines exhibited morphological defects, including abnormal rachis architecture, reduced cross-sectional surface area of germline compartments, and displacement of compartments from their normal positioning within the germline. *ced-3(lf)* animals yielded abnormally small embryos; these small embryos had decreased embryonic viability. These findings provide insights into the functional relevance of apoptosis and germline morphology for fertility.

## RESULTS

### *ced-3(lf)* hermaphrodites exhibit brood size and germline architecture defects that intensify over the reproductive lifespan

Apoptosis has been linked to maintenance of germline architecture and disassembly in aged hermaphrodites exhausted of sperm (de la Guardia et al., 2016; Gumienny et al., 1999). Additionally, loss of apoptosis was also linked to increases in age-dependent embryonic lethality in mated virgin females (Andux and Ellis, 2008). Finally, slight decreases in embryonic viability and total brood size have also been observed in apoptosis defective animals (Ellis and Horvitz, 1986; Hengartner et al., 1992). However, no cell biological characterization of the origin of these defects in fertile hermaphrodites has been carried out to date. We used the apoptosis-defective *ced-3* loss-of-function mutant *ced-3*(*n717*) [hereafter *ced-3(lf)*] (Ellis and Horvitz, 1986) to recapitulate previously reported defects in hermaphrodite fertility (Ellis and Horvitz, 1986; Hengartner et al., 1992). The total number of embryos laid (brood size) within the fertile period (the 60 h following L4) was significantly lower for *ced-3*(*lf*) hermaphrodites than wild-type control animals (Fig. 1A). Given the age-dependent effect of loss of apoptosis on embryonic viability in mated virgin females, we tested whether defects in fertility showed an age-dependent progression in severity in fertile hermaphrodites as well. We measured the average brood count in three distinct time windows: 0-24, 24-44 and 44-60 h post L4. At 24 h, control and *ced-3(lf*) brood sizes were comparable; however, the mutants failed to maintain a normal brood size from that point onwards (Fig. 1B). The reduction in brood size, and therefore germline function, in *ced-3(lf)* animals suggested that apoptosis is required for normal oogenesis.

**Fig. 2.**
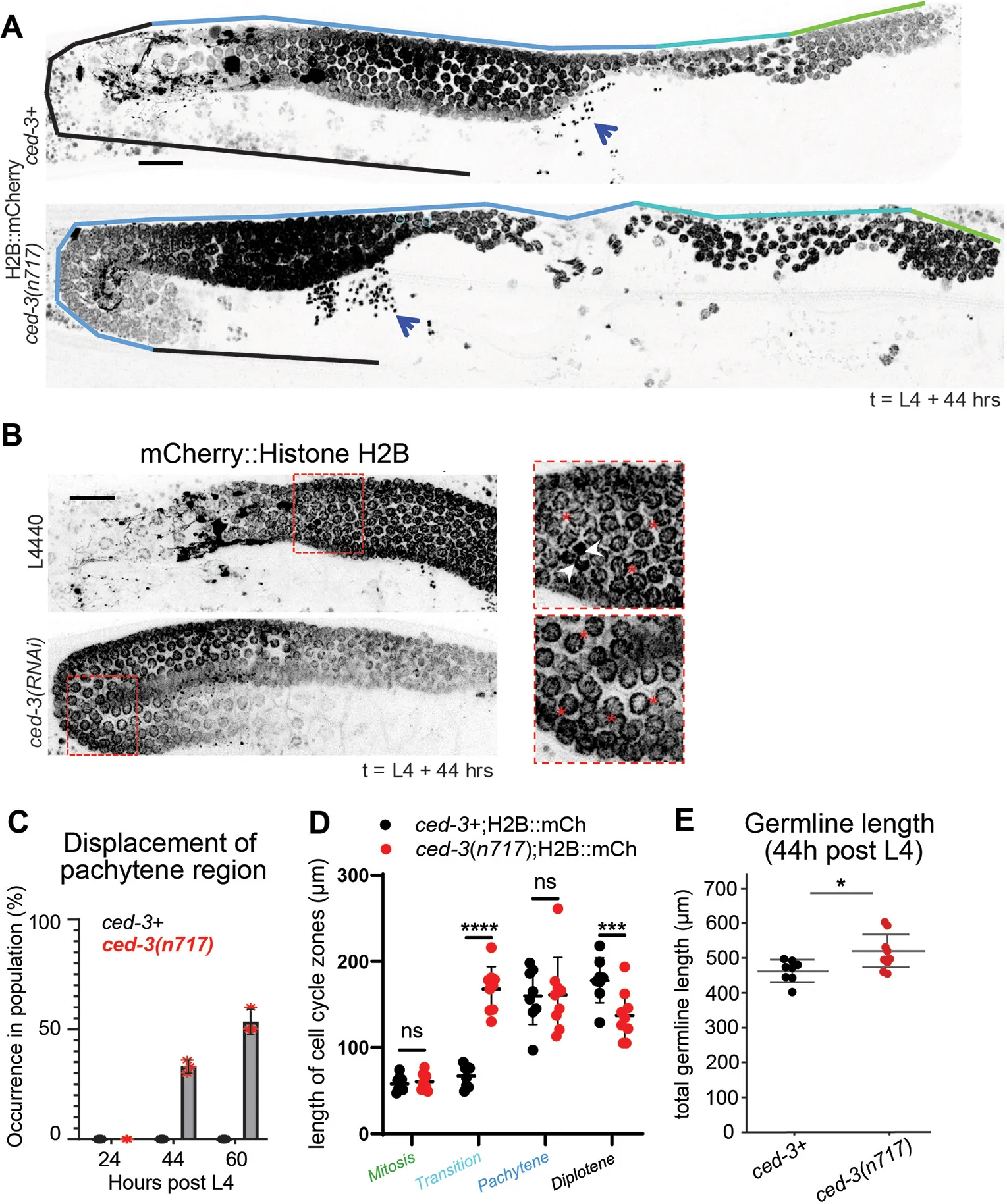
***ced-3(lf)* mutants compensate for excess germ cells by delaying cell cycle progression and cellularization.** (A) Images of the organization of germline nuclei (H2B::mCherry) in the indicated genotypes at 44 h post L4, with colored lines indicating cell cycle stages of germline nuclei based on chromatin appearance. Cyan circles highlight intermingled transition state nuclei within pachytene. Blue arrowheads point to sperm DNA marked by H2B::mCherry fluorescence. (B) Maximum projection images of germ compartment nuclei (H2B::mCherry) in the experimental conditions indicated. Pachytene nuclei (red dashed box). Within the enlarged images of nuclei (insets), apoptotic nuclei in an L4440 control germline (white arrows), pachytene nuclei displaced in *ced-3*(*RNAi)* conditions (red asterisks). (C) Quantification of displacement of pachytene nuclei in the indicated genotypes. Data represent three independent experiments (*n*=10 germlines per genotype). (D) Quantification of the percentage of the rachis occupied by mitotic, transition, pachytene and diplotene/diakinesis nuclei in *ced-3*+ and ced*-3*(*lf*) germlines, at 44 h post L4. *n*≥8 germlines per genotype analyzed. ****P≤0.0001, ***P≤0.001 (two-way ANOVA with Tukey's multiple comparisons test). ns, not statistically significantly different. (E) Measurements of total germline length from distal tip to the spermatheca in control (MDX155; *n*=8) and *ced-3* mutant (MDX187; *n*=9) animals at 44 h post L4. *P<0.05 (two-tailed Student's *t*-test). Error bars indicate mean±s.d. in all panels unless specified otherwise. Scale bars: 20 μm.

Various aspects of germline morphology are correlated with successful oogenesis (Green et al., 2011). Young adult *ced-3(lf)* mutant germlines are reported to be morphologically indistinguishable from wild type, while post-fertility aged germlines (72 h post L4) show defects in germline architecture (Das et al., 2020; Gumienny et al., 1999). To test whether *ced-3*(*lf*) fertility defects in older fertile hermaphrodites were due to changes in germline morphology, we examined germlines of animals expressing fluorescently labeled anillin-1 (ANI-1), a cortical scaffolding protein (Maddox et al., 2005; Rehain-Bell et al., 2017). ANI-1 localizes to the germline cortex, rachis bridges, oocyte boundaries, and rachis lining (Amini et al., 2014; Rehain-Bell et al., 2017), also illustrated in the schematic (Fig. 1C). In accordance with others' and our observation that *ced-3(lf)* mutants generated a normal number of progeny in the first 24 h of adulthood, germline architecture was largely normal 24 h after L4 (Das et al., 2020) (Fig. 1D). However, 20 h later (44 h into adulthood), *ced-3*(*lf*) mutant animals laid significantly fewer progeny (Fig. 1B), and germlines were morphologically abnormal (Fig. 1E,F). In control germlines, developing oocytes were arranged in a single row throughout the spermatheca-proximal germline (Fig. S1A), but in *ced-3*(*lf*) germlines oocytes in the proximal germline were often abnormally small and failed to form a single row (‘clustered’), abnormally numerous and occupying a single row of short cylinders (‘stacked’), or both (Fig. 1E,F). These morphological defects were observed in both the anterior and the posterior germlines. Since accumulation of ‘stacked’ oocytes can occur when hermaphrodites are depleted of sperm (Tolkin and Hubbard, 2021), we assessed the time course of the appearance of the various phenotypes. *ced-3(lf)* germlines first exhibited stacked oocytes, then clustered, and a combination of both, as animals progressed from 24 to 44 h post L4 (Fig. 1F). Approximately 5% of control animals exhibited stacked oocytes in the proximal germline at 44 h post L4, but no other defects were observed (Fig. 1F). The percentage of *ced-3(lf)* mutant germlines that appeared normal decreased over time to ∼33% at 44 h post L4 (Fig. 1F). These germline morphology defects did not stem from sperm exhaustion, as at 44 h post L4, like control animals, *ced-3*(*lf*) animals' spermathecas retained sperm (Fig. 2A). Furthermore, introduction of sperm by mating *ced-3(lf)* adult hermaphrodites with males wild type for *ced-3* did not restore germline structure (Fig. S2C). These observations collectively highlight the importance of apoptosis in ensuring normal germline function and morphology.

### Loss of apoptosis perturbs oogenic cell cycle progression

Oogenesis largely depends on the progression of germline compartments from mitosis into, and through the stages of, prophase of meiosis I (Kim et al., 2013). We compared the nuclear progression through oogenesis in control and *ced-3(lf)* germlines by assessing DNA morphology using an mCherry-tagged histone variant (H2B::mCherry) as defined previously (Crittenden et al., 1994; Albert Hubbard, 2007; Jaramillo-Lambert et al., 2007; Lints and Hall, 2009). In control animals, compartments immediately preceding the bend were in pachytene, and compartments around the bend and in the proximal germline were in first diplotene and then diakinesis; groups of compartments in each stage were distinct and did not intermingle (Fig. 2A,B). In *ced-3(lf)* animals, the distended histone-rich structures of engulfed apoptotic corpses undergoing digestion were absent from the surrounding sheath cells (Fig. 2A). In *ced-3(lf)* germlines, compartments in pachytene extended around the bend (Fig. 2A). This phenomenon, as well as clustering and stacking of compartments in the proximal germline, was also observed in animals depleted of CED-3 by RNAi (Fig. 2B). Displacement of the pachytene region around the germline bend was first observed 44 h after L4 and was more prevalent with time (Fig. 2C). The number of compartments in each stage (and thus the length of the germline they occupy) reflects the duration of each stage. Measurements of the proportion of the germline occupied by mitotic, transition, pachytene and diplotene/diakinesis nuclei revealed that, in comparison to control germlines, the transition zone was extended in *ced-3(lf)* germlines (Fig. 2A,D). Comparable results were obtained specific to the transition zone when CED-3 was depleted in animals expressing REC-8::GFP, which served as a landmark for discerning mitotically dividing germ cell nuclei when compared with L4440 treated controls (Fig. S2A). However, in the RNAi depleted animals, the mitotic zone was slightly shorter than in L4440 animals (Fig. S2B). The total length of the germline was accordingly significantly longer in *ced-3(lf)* animals compared to controls (Fig. 2E). Taken together, our findings suggest that loss of apoptosis leads to delays in cell cycle progression and leads to significantly longer germlines.

### *ced-3(lf)* germlines have abnormal rachis and germline compartment architecture

**Fig. 3.**
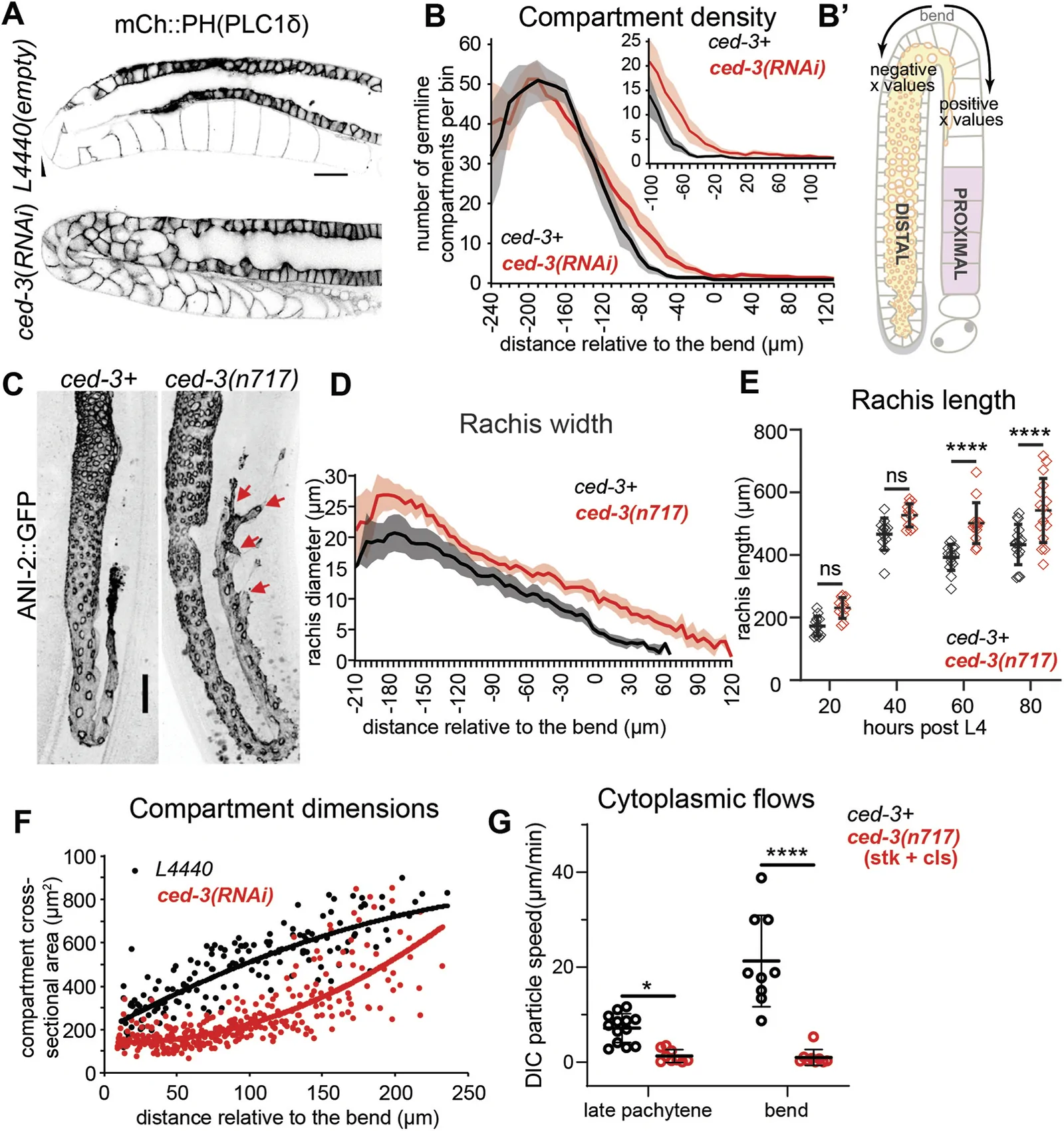
***ced-3(lf)* germlines have abnormal rachis and germline compartment morphology.** (A) Representative images of membrane (mCherry::PH, MDX63) localization used for the quantifications in B and E. Animals were fed with RNAi bacteria for 44 h; L4440: empty vector (top); *ced-3(RNAi)* (bottom). (B) Average number of compartments within 10 μm bins of the germline, at positions relative to the bend in control (*n*=14) and *ced-3(RNAi)* (*n*=20) animals. Shaded areas represent 95% confidence intervals. Inset shows magnified view of part of the graph. (B′) Schematic depicting the position of positive and negative germline distances (*x*-values) from the bend, represented in B, D and E. (C) Representative images (maximum projections) of the rachis (ANI-2::GFP) in the indicated genotypes at 44 h post L4. Red arrows highlight abnormal bifurcations/protrusions. (D) Quantification of rachis diameter throughout the germline (from distal tip to cellularization) in the indicated genotypes. *n*=14 animal germlines per genotype. Shaded areas represent 95% confidence intervals. (E) Quantification of germline length in synchronized populations in the indicated genotypes. *n*≥13 germlines per condition, and time post L4 indicated. Error bars represent mean±s.d. ****P≤0.0001 (two-way ANOVA with Šídák's multiple comparisons post-hoc test). ns, not significant (20 h, *P*=0.092; 40 h, *P*=0.062). (F) Measurement of the cross-sectional area of germline compartments in control and CED-3-depleted animals. *n*=15 control (L4440) and 10 *ced-3(RNAi)* germlines. (G) Quantification of cytoplasmic flow speeds in the late pachytene and germline bend/loop regions in the indicated genotypes, at 44 h post L4. stk+cls, stacked and clustered morphology. One or two particles were tracked for *n*=8 germlines per genotype indicated. ****P≤0.0001, *P=0.0463 (two-way ANOVA with Tukey's multiple comparisons test). Scale bars: 20 μm.

As oogenic compartments proceed through prophase of meiosis I, they gradually mature and enlarge, while remaining connected with the syncytium. Given the gross defects in germline morphology reported above, we next sought to understand how the *ced-3(lf)* effect on germline cell cycle progression relates to syncytial architecture. We have already established, along with others, that CED-3 depletion was sufficient to block apoptosis and caused decreased fertility (Fig. S1A). Because of the abnormal morphologies observed, we were unable to use mNG::ANI-1 to delineate cell boundaries in the mutant. We therefore used an mCherry-tagged membrane-binding probe to assess germline morphology more accurately. We depleted CED-3 by RNAi and documented germline morphology 44 h after L4, when significant germline architecture defects were observed. Germline compartments of control animals formed a single row around the germline bend and throughout the proximal germline. By contrast, these domains in *ced-3(lf)* animals were occupied by smaller, often irregularly shaped compartments (Fig. 3A). We then quantified the number of germline compartments in each 10 μm bin throughout the germline of both control and *ced-3(lf)* animals. The number of germline compartments per unit length was consistently higher in *ced-3*(*lf*) mutants than in controls starting at 100 μm distal to the bend in the germline, which in wild-type worms corresponds to mid to late pachytene, the region where most apoptosis occurs (Fig. 3B,B′).

The extension of the pachytene zone around the bend and overall longer germlines in *ced-3(lf)* suggested that the rachis is longer in the absence of apoptosis. However, in some conditions with abnormally small and inviable oocytes, the rachis collapses (closes) prematurely (Green et al., 2011; Maddox et al., 2005). To test how changes in germline compartment number in *ced-3(lf)* germlines related to abnormal rachis morphology, we measured rachis diameter throughout the germline using ANI-2::GFP, which specifically marks the rachis lining, in control and *ced-3*(*lf*) animals 44 h post L4. In *ced-3*(*lf*) germlines, as in controls, the rachis steadily narrowed from pachytene to cellularization but was wider along its entire length in *ced-3*(*lf*) animals (Fig. 3C,D). Furthermore, the rachis was significantly longer in *ced-3(lf)* animals 44 h or more beyond L4 (Fig. 3D,E). The proximal part of the germline was also significantly extended in the germline of *ced-3*(*lf*) animals (Fig. S1D). Interestingly, most (45/50) of the *ced-3* mutant germlines displayed a bifurcated rachis at 44 h post L4, compared to none of the control animals (*n*=52; Fig. 3C, red arrows).

To determine whether the changes in germline architecture [increased density of germline compartments and increased rachis diameter and length in *ced-3(lf)* mutants] translated to changes in germline compartment and oocyte size, we next measured the cross-sectional area of individual compartments proximal to the bend at 44 h post L4 in both control and *ced-3(RNAi)* worms. In CED-3-depleted animals, germline compartments past the germline bend were smaller compared to controls (Fig. 3F). Thus, despite being abnormally long, *ced-3(lf)* germlines contain an unusually large number of abnormally small compartments and oocytes. Extending the distal germline by depleting CCDC-55 (Kovacevic et al., 2012) did not rescue the architectural defects and small oocytes of *ced-3(lf)* germlines and caused pleiotropic morphological defects (data not shown).

Germline compartment enlargement is thought to occur via cytoplasmic flows from the syncytial core (the rachis); compartments at the germline bend and in the proximal germline are net acceptors of cytoplasm (Chartier et al., 2021; Wolke et al., 2007). To determine how compartments with sustained syncytial connection were abnormally small, we measured cytoplasmic flow speed. Cytoplasmic flow speed had been reported to be normal in *ced-3*(*lf*) early adults (12-24 h post L4) (Wolke et al., 2007) (Fig. S1E). Since we observed smaller ‘stacked and clustered’ oocytes as *ced-3(lf)* animals aged, we measured cytoplasmic flow speeds 44 h post L4. In control germlines, the average flow speeds in late pachytene and around the germline bend were 12 μm/min and 21 μm/min, respectively (Fig. 3G, Movie 1). By contrast, in *ced-3(lf)* animals with germlines exhibiting the ‘stacked and clustered’ morphology, flows were 0.6 μm/min in pachytene and 0.4 μm/min at the bend (Fig. 3G, Movie 2). These results agree with previous reports on the importance of cytoplasmic flows for oocyte expansion (Wolke et al., 2007). The rate at which cytoplasm flows into oocytes has been reported to be mainly regulated via actomyosin (Nadarajan et al., 2009; Wolke et al., 2007). In agreement, non-muscle myosin II (NMY-2) was less abundant on germline cytoplasmic bridges in *ced-3(lf)* germlines (Fig. S1F), similar to previous reports (Kohlbrenner et al., 2024).

Together, these findings suggest that apoptosis is required for oogenesis by maintaining normal germline architecture during oogenesis.

### Apoptosis promotes embryonic viability by ensuring oocyte size

**Fig. 4.**
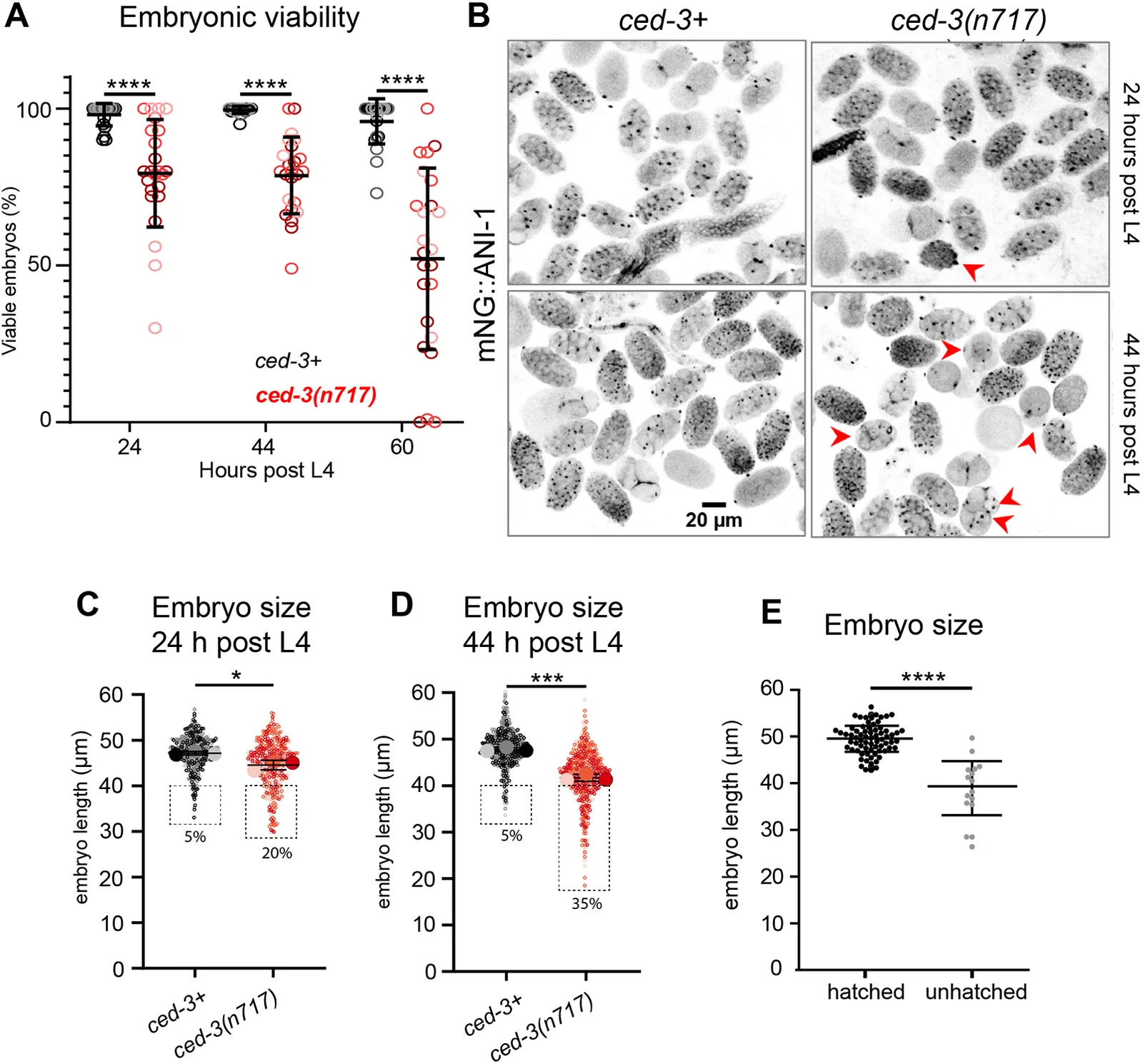
**Apoptosis promotes embryonic viability by ensuring oocyte size.** (A) Embryonic viability of progeny of control and *ced-3(n717)* animals at 0-24 h, 24-48 h and 48-60 h post L4. Data points represent individual worms from three independent experiments (*n*=24 animals per genotype). ****P<0.0001 (ANOVA followed by Šídák's post hoc analysis). Error bars represent mean±s.d. (B) Representative images of embryos extruded from animals at 24 and 44 h post L4 in the indicated genotypes. Red arrowheads point to abnormally small embryos. Scale bar: 20 μm. (C,D) Quantification of embryo length in the listed genotypes for animals at 24 h (C) and 44 h (D) post L4. Percentage of embryos smaller than 40 μm long are shown and highlighted by dashed boxes. Three independent experiments, each with *n*≥14 animals per genotype. *P=0.0361, ***P=0.0009 (Welch's two-tailed *t*-test). Error bars represent mean±s.d. (E) Quantification of embryo length and viability from overnight time-lapse imaging of embryos pooled from *ced-3*(*lf*) animals at 44 h post L4. *n*=15 animals. ****P<0.0001 (two-tailed Student's *t*-test).

It has been reported that loss of apoptosis leads to a decrease in embryonic viability (Ellis and Horvitz, 1986; Hengartner et al., 1992). This could be due to the failure to remove germline compartments with DNA damage and multinucleation, or due to defects in embryo size as a consequence of defects in germline architecture, or a combination of both. To explore these possibilities, we assessed the viability of embryos produced by control and *ced-3*(*lf*) animals over time. Compared to controls, the viability of *ced-3(lf)* mutant embryos was 20% lower, even in early fertility (0-24 h post L4) when only mild germline architecture defects were observed. This likely represents the baseline of embryonic lethality due to the fertilization of oocytes with DNA damage or multinucleation (Fig. 4A; Raiders et al., 2018). In the next 24-h time window (24-44 h post L4), at the end of which germline architecture defects are readily observed, embryonic lethality was the same as in earlier stages. By contrast, at 44-60 h post L4, embryonic lethality was significantly higher, reaching nearly 50% (Fig. 4A). These data suggested that at least some of the observed defects in embryonic viability are due to defects in germline architecture.

Given that smaller embryos are less likely to be viable (Hubatsch et al., 2019; Maddox et al., 2005; Tscherner et al., 2021) and that one of the major defects we observe in *ced-3(lf)* animals is a significant reduction in germline compartment and oocyte size (Fig. 2E,F), we examined whether the increase in embryonic lethality observed 44 h and longer after L4 correlated with an increase in the population of small embryos. We measured embryo size by extruding embryos from gravid hermaphrodites at 24 and 44 h post L4, to assess embryo populations in the 24-44 and 44-60 h post L4 time windows, respectively. Compared to wild-type embryos, which appeared normal in size, many *ced-3(lf)* embryos were abnormally small at 44 h post L4 (Fig. 4B). Specifically, ∼35% of *ced-3(lf)* embryos were at a length of 40 μm or less compared to ∼5% of control embryos. Small embryos were also produced, rarely, by *ced-3(lf)* animals at 24 h post L4 (Fig. 4C,D), possibly reflecting how some *ced-3*(*lf*) animals displayed morphological defects of stacking and clustering even at 24 h post L4 (Fig. 1F).

To confirm that small *ced-3(lf)* embryos are indeed those that fail to develop into viable worms, we dissected *ced-3*(*lf*) animals at 44 h post L4 to observe their embryos by time-lapse imaging for 24 h at 20°C. Whereas wild-type *C. elegans* complete embryogenesis and hatch within 12 h of fertilization at 20°C (Hall et al., 2017), 19% of *ced-3*(*lf*) embryos failed to hatch (Fig. 4E) even after 24 h. We measured the size (anterior-posterior axis length) of each embryo and scored whether hatching occurred. Embryos that hatched were on average 49 μm in length while the average length of unhatched embryos was 39.5 μm. All *ced-3*(*lf*) embryos over 40 μm in length hatched; all those below 40 μm in length were unable to complete embryogenesis (Fig. 4E). Some normal-sized embryos died as well, potentially reflecting the subset of embryos dying as a consequence of DNA damage or multinucleation (Fig. 4E). This suggests that increased embryonic lethality in *ced-3(lf)* 44 h post L4 animals is indeed caused by reduced embryo size, which itself arises from defects in germline architecture. These findings indicate that, in addition to ensuring embryonic viability via DNA damage surveillance in the germline, apoptosis also contributes to embryonic fitness by maintaining germline morphology, to accomplish the production of viable progeny.

## DISCUSSION

We investigated how apoptosis promotes fertility and oocyte quality during oogenesis in *C. elegans* using loss-of-function perturbations of the germline apoptosis caspase CED-3. We find that worms lacking apoptosis exhibit fertility defects that become more pronounced with age. The observed reduction in brood size correlates with an age-dependent increase in defects in germline architecture. These defects included an increase in germline compartment number, an increase in rachis cross-sectional area, and a decrease in germ cell size proximal to the bend. These defects may be partially counterbalanced by the observed increase in the duration of the transition zone stage and extension of the proximal rachis to delay cellularization. The appearance of architectural defects coincided with an age-dependent increase in embryonic lethality that likely reflects fertilization of smaller oocytes that fail to develop.

Our time course analysis of brood size, embryonic viability, and germline architecture allowed us to reconcile seemingly contradictory results from previous studies that reported no significant germline defects in young adult animals (Das et al., 2020; Gumienny et al., 1999) and reduced brood size and embryonic viability, as well as significant germline architecture defects, in hermaphrodites exhausted of sperm (Ellis and Horvitz, 1986; Gumienny et al., 1999; Hengartner et al., 1992). We propose that in the absence of apoptosis, *C. elegans* animals partially compensate for the increase in total germline compartments that are no longer removed during pachytene by delaying exit of germ cells from the transition zone and extending the proximal rachis. Indeed, normal cell-cycle exit of germ cell compartments in the germline is defective in *ced-3*(*lf*) animals; however, how this defect arises is not understood. Since *ced-3*(*lf*) has more compartments, these shifts could be due to the inability of the germline to remove pachytene compartments, which may slow down transition exit, but this requires further exploration. Potentially, extension of the area occupied by transition zone compartments delays the onset of diplotene and diakinesis, when compartments significantly increase in size (Rehain-Bell et al., 2017), thus slowing enlargement and cellularization of oocytes. These compensatory mechanisms may be sufficient to ensure normal oocyte morphology and number in young adults (Das et al., 2020; Gumienny et al., 1999), thus preserving the effect on overall brood size and embryonic viability. As the animals age, however, the germline loses the capacity to compensate for the excess compartments, and defects in germline architecture, fecundity, and embryonic viability result (as reported by Ellis and Horvitz, 1986; Hengartner et al., 1992). An open question in this context remains: how is this spatial adaptation of meiotic progression coordinated with cell cycle timing? A prime candidate for this coordination is ERK-1,2/MPK-1 signaling, which is important for proper germline architecture, oocyte size, and progression into both pachytene and diplotene (Church et al., 1995; Lee et al., 2007a,b; Robinson-Thiewes et al., 2021). Furthermore, this signaling pathway has been implicated in regulating oocyte size and apical constrictions of apoptotic germ cells (Kohlbrenner et al., 2024). Understanding how ERK/MAPK signaling is affected by increased germline compartment number, and how defects in this signaling pathway affect germline architecture in apoptotic defective animals, will be the focus of future inquiry.

CED-3 activity is regulated by the BCL-2-like protein CED-9, which is proposed to limit excessive apoptosis through mechanisms that remain unclear (Hengartner et al., 1992), and by the upstream BH3-only protein EGL-1, which modulates CED-3 function in somatic tissues but not in the germline (Gumienny et al., 1999). Thus, CED-3 regulation in the germline differs fundamentally from its control in the soma and remains poorly understood. Although apoptosis is known to eliminate germ cells with defective nuclei, such as those carrying DNA damage, our findings indicate that it is also required to maintain germline architecture and support oogenic function. Over half of the germ cells made in *C. elegans* germlines are removed by apoptosis (Gumienny et al., 1999; Jaramillo-Lambert et al., 2007). Beyond removing abnormal binucleate compartments (Raiders et al., 2018; Robinson-Thiewes et al., 2021), CED-3 can also eliminate otherwise normal germ cell compartments in response to other factors during oogenesis in *C. elegans*, although the mechanism underlying this process is unknown (Ito et al., 2010; Salinas et al., 2006). Defining how proteins act differentially in somatic versus germline contexts will be essential for understanding how CED-3 contributes to oogenesis. Notably, ERK/MPK exhibits germline-specific and somatic isoforms (Kohlbrenner et al., 2024; Rutkowski et al., 2011), raising the possibility that CED-3 may likewise have distinct isoforms or be regulated by MPK signaling, a question that remains unresolved. Apoptosis has been shown to play a plethora of roles during development and tissue homeostasis in animals (Fuchs and Steller, 2011; Ghose et al., 2018; Voss and Strasser, 2020). It can act as a quality control mechanism removing cells bearing defects caused by environmental stress, infection or mitotic errors (Elmore, 2007; Luo et al., 2010; Norbury and Hickson, 2001; Raiders et al., 2018). However, apoptosis also plays vital roles in eliminating specific populations of otherwise healthy cells to support tissue morphogenesis and architecture. The role of apoptosis in removing germline compartments containing DNA damage caused by environmental stress or meiotic failures is well documented in *C. elegans*; the persistence of such defective oogenic compartments is likely reflected in the ∼20% of baseline embryonic lethality observed at all timepoints in *ced-3(lf)* worms (Andux and Ellis, 2008; Raiders et al., 2018). The fact that loss of apoptosis also leads to significant changes in germline architecture, associated with defects in fertility and embryonic viability, suggests that the role of apoptosis in the germline extends beyond ensuring nuclear quality control, to an important contribution to germline architecture. Our findings suggest that these processes collaborate to maximize the number of viable fertilized embryos. How germline architecture and, by extension, germline compartment volume, is regulated is less well understood, but it is generally assumed that a delicate force balance between contractility of the actomyosin cortex that lines the rachis, and cytoplasmic streaming within this common cytoplasm, is required for maintaining proper germline architecture and for regulating oocyte size (Nadarajan et al., 2009; Priti et al., 2018; Rehain-Bell et al., 2017; Wolke et al., 2007). Indeed, the increase in total compartment number due to a lack of apoptosis in *ced-3(lf)* animals leads to defects in germline architecture, reduced cytoplasmic streaming, and thus oocyte size. We also note the increased nuclear localization of ANI-1 in ‘stacked’ or ‘clustered’ oocytes in animals lacking apoptosis; this change in the localization of a cortical contractility protein may affect the force balance among germline compartments. These defects are probably due to a combination of delayed entry into pachytene, postponed oocyte enlargement and thus less time for this process before detachment from the common cytoplasm during cellularization, and/or spatial limitation of oocyte enlargement due to severely reduced streaming in response to progressive overcrowding of the germline with compartments that ordinarily would be removed by apoptosis. Future work will address how the cytoskeletal mechanisms of germline architecture maintenance relate to apoptotic dynamics and/or the abundance of compartments.

Another developmental window in which apoptosis has been implicated in germline function is in aging worms past the normal window of fertility. As hermaphrodites age and sperm are depleted, the germline progressively atrophies (Kocsisova et al., 2025, 2019). This process is accelerated by apoptosis (de la Guardia et al., 2016). Feminized worms (that do not produce sperm) aged for various durations before being provided with sperm via mating exhibited an age-dependent reduction in embryonic viability that was significantly enhanced in the absence of apoptosis (Andux and Ellis, 2008). Interestingly, in this context, as in our work, increased embryonic lethality correlated with decreased oocyte size, suggesting that both during the time-window of normal fertility and in aged virgin females similar mechanisms for regulating oocyte quality by maintaining germline architecture are at work. These similarities raise the possibility that another biological role for germline apoptosis is to manage germline architecture and oocyte production in response to sperm availability, executing the dismantling of the germline, redirecting metabolic resources in the absence of sperm, and promoting proper germline architecture and oocyte size when sperm are provided by mating. It will be interesting to investigate in detail how germline architecture evolves in these conditions.

## MATERIALS AND METHODS

### *C. elegans* strains and culture

*C. elegans* strains were maintained at 20°C using standard procedures (Brenner, 1974). The strains used in this work are listed in Table S1. MDX129 was generated by crossing MDX40 and MT1522. MDX155 was obtained by crossing UM208 and KLG049. MDX187 was created by crossing MDX155 and MT1522. MDX63 was generated by crossing MDX29 and LP193. KLG049 was a generous gift from Kacy Gordon (University of North Carolina at Chapel Hill, NC, USA) and generation of the allele was initially described by Roy et al. (2018).

### RNA interference (RNAi)

RNAi was performed by feeding HT115 *Escherichia coli* bacteria expressing double-strand RNA at 20°C for 24 h as described previously (Kamath and Ahringer, 2003; Kamath et al., 2003). Individual bacterial clones were obtained from an RNAi library (gift from Bob Goldstein, University of North Carolina at Chapel Hill, NC, USA) (Kamath and Ahringer, 2003). The targets of clones used in this study were confirmed by sequencing. RNAi treatments were continuously maintained from L4 through 24-, 44- and 60-h durations wherever used. Worms were transferred to new RNAi plates daily to avoid overcrowding of newly hatching embryos for 44-h and 60-h treatments.

### Synchronizing *C. elegans*

*C. elegans* synchronization was performed as previously described (Perry et al., 2023). L1 animals were monitored until L4 when they were collected and mounted for imaging of their germlines at 24, 44 and 72 h post L4.

### Imaging *C. elegans* germlines and embryos

#### Imaging dish preparation

A mixture of 1% agarose and 5 mM levamisole in 50 ml of M9 buffer was heated until the agarose was dissolved, then the mixture was briefly cooled, and poured, in ∼3 ml quantities into 35 mm Mattek coverslip-bottom imaging dishes (Mattek Corporation, P35G-0.170-14-C). Dishes were cooled for 30 min in a hood, wrapped with parafilm, and stored at 4°C for use within 4 months.

#### Live mounting for imaging

For all imaging experiments, live hermaphrodites were picked at L4 and mounted into 35 mm Mattek agarose imaging dishes. With the 1% agar pad in a prepared imaging dish gently lifted, 0.8 μl of 5 mM levamisole in M9 buffer was pipetted onto the coverslip. For still imaging of live animals, five to seven live hermaphrodites were gently placed onto the 0.8 μl spot using a platinum pick, and, after immobilization, positioned with an eyelash tool. The agar pad was gently repositioned in contact with the plate bottom to hold animals in place for live imaging by gentle compression.

#### Fluorescence microscopy

Fluorescence imaging of live worms was performed using a 60×, 1.27 NA Nikon Water Immersion Objective on a Nikon A1R microscope with a Gallium arsenide phosphide photo-multiplier tube (GaAsP PMT) detector using NIS-elements. Approximately 35 optical sections, separated by 1 μm, were collected for the fluorescent images in Figs 2 and 3. All fluorescence imaging was performed by this method unless specified. The still images of germlines in Fig. 1D,E and Fig. S1B were collected with a 10×, NA 0.30 objective on a Nikon A1R, 5 μm *z*-steps, and around seven total steps collected (only the middle section is shown).

#### Imaging embryos

L4 animals of the indicated genotypes were grown for 24 or 44 h on NGM plates. Animals were picked into a 2-5 μl droplet of M9 on the coverslip of a premade Mattek imaging dish with a 1% agarose pad (lift off as described in the ‘Live mounting for imaging’ section). A hypodermic needle was used to cut gravid adults near the uterus to release embryos into M9 buffer (0.85 M Na_2_HPO_4_, 0.4 M KH_2_PO_4_, 0.3 M NaCl, 1 mM MgSO_4_). An eyelash tool was used to position embryos horizontally on Mattek imaging dishes. The 1% agar pad was slowly and gently re-positioned over the embryos and dishes were mounted on the stage for imaging. Single frame images were acquired with a 10×, NA 0.30 objective on a Nikon A1R.

#### Differential interference contrast (DIC) time-lapse imaging

Worms were mounted as described in the ‘Imaging embryos’ section. Time course images of N2 and MT1522 germlines were obtained on a Nikon Eclipse Ti2-FP widefield microscope with a Hamamatsu Orca FusionBT camera. Single-plane images were captured using a 60×1.20 NA water immersion objective, with polarized light. Worm germlines were brought into focus where cytoplasmic particles were visible prior to imaging, and acquisitions were obtained at 2-s intervals.

### Brood size analysis

Ten to twelve N2 and MT1522 hermaphrodites at L4 were placed onto NGM plates seeded with OP50 *E. coli*. The number of embryos laid by each animal was counted in the time windows of 0-24 h, 24-48 h and 48-60 h, consecutively. Animals were moved to new NGM to fresh plates at each timepoint. The total number of hatched and unhatched embryos per adult at each timepoint were combined to determine the total 60-h brood count. For measuring embryonic viability, we allowed embryos to hatch and counted the number of live progeny for each adult ∼40 h after assessing brood size. Embryonic viability was calculated at each timepoint as follows:

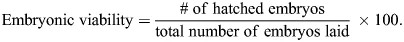


### Image analysis

Image analysis was performed using Fiji/Image J software (Schindelin et al., 2012). Unless specified otherwise, Maximum projections of *z*-sections were generated to show cortices, intercellular bridges, rings, rachis and oogonia compartments starting at the distal tip through to the proximal germline arm.

#### Assessment of cell-cycle zones

The cell-cycle zones of oogonia were discerned based on chromatin morphology as previously described (Albert Hubbard, 2007). These were determined by scrolling through *z*-sections and analyzing chromatin shape and structure through 1 μm *z*-steps of germline images for each experimental condition, with the operator unaware of experimental groupings. Zones were defined by previously described morphology (Lints and Hall, 2009) as follows: mitotic (distal tip nuclei devoid of crescent-shaped histones); transition (chromatin occupying a crescent of the nucleus devoid of pachytene); pachytene (‘bowl-of-spaghetti’ appearance nuclei devoid of diplotene nuclei); diplotene/diakinesis (diplotene and diakinesis nuclei including bivalents).

#### Intensity measurements

Average fluorescence intensity was obtained in Fiji (ImageJ) by collecting the intensity of four 1 pixel×1 pixel dots positioned on individual rings. Each of these four dots were averaged for each ring measured, and the mean intensity reported per x position on the germline. The position of these dots (x) was marked by using the germline bend as the reference point. All positive x (position) values are representative of points before the bend (distal), position 0 marks the bend, and all negative values represent positions after the bend (proximal).

#### Quantifying cytoplasmic flow speed

Cytoplasmic speed was analyzed by tracking DIC particles in late pachytene and within the germline bend/loop in ImageJ (Fiji). Particle tracks were obtained from each germline region using the ImageJ ‘reslice’ tool to generate kymographs. The slope of these tracks was then calculated in Excel using *x* and *y* positions over time per track, and reported as DIC particle flow speed or cytoplasmic flow speed.

### Germline rachis measurements

Rachis width was determined manually using Fiji (Schindelin et al., 2012) by measuring rachis diameter in various parts of the germline using ANI-2::GFP (fluorophores and strains used) to mark the outline of the rachis lining. These measurements were used to calculate cross-sectional rachis area by approximating the rachis cross-sectional area to a circle using a custom R script (available upon request).

Germline compartment number was determined using automated segmentation of germline nuclei in the distal part of the germline before the bend in strains expressing H2B::mCherry (MDX 155 and MDX187) using Cell-ACDC (Padovani et al., 2022; Pachitariu and Stringer, 2022). Segmentation results were curated manually to remove misannotated areas and include nuclei missed by the segmentation. For parts of the germline proximal to the bend, the segmentation failed and nuclei were identified manually using Fiji.

To measure germ cell cross-sectional area, germ cell compartments were approximated to be rectangles in the MDX63 worm strain expressing mCherry::PH(PLC1-delta1) to label germ cell membranes. Worms were treated with control or *ced-3*(*RNAi*) for 44 h and the length and width of the germ cell compartment were measured by placing a point on apical and basal regions as well as each lateral membrane of a germ cell, respectively, using the multipoint tool in Fiji. Cross-sectional area was estimated by calculating the distance between the two pairs of points and using these values as width and length measurements to calculate the area of the rectangle using a custom Python script.

For all measurements, the basal tip of the bend of the germline was used as a reference point to position the individual measurements along the length of the germline, by calculating the distance between the reference point and the position of the center of the measured objects.

Measurements distal to the bend were assigned a negative positional value while proximal measurements were assigned positive positional values, in order to be able to plot the measurements as developmental time courses in the same graph. For graphical depiction of developmental time courses of both rachis area and germ cell compartment number, measurements were binned into 10 μm bins based on their position relative to the reference point in the germline bend using custom scripts in R and Python. Mean values and 95% confidence intervals were calculated and plotted for each bin using Microsoft Excel.

## Supplementary Material



10.1242/develop.205442_sup1 Supplementary information
