# Supplementary material for: Apoptosis promotes fertility in Caenorhabditis elegans by maintaining functional germline morphology
Source: Development. 2026 Aug 13;153(15):dev205442. doi: 10.1242/dev.205442 (PMC13535102; doi:10.1242/dev.205442)
Supplement: Supplementary file 1 [file develop-153-205442-s1.pdf]

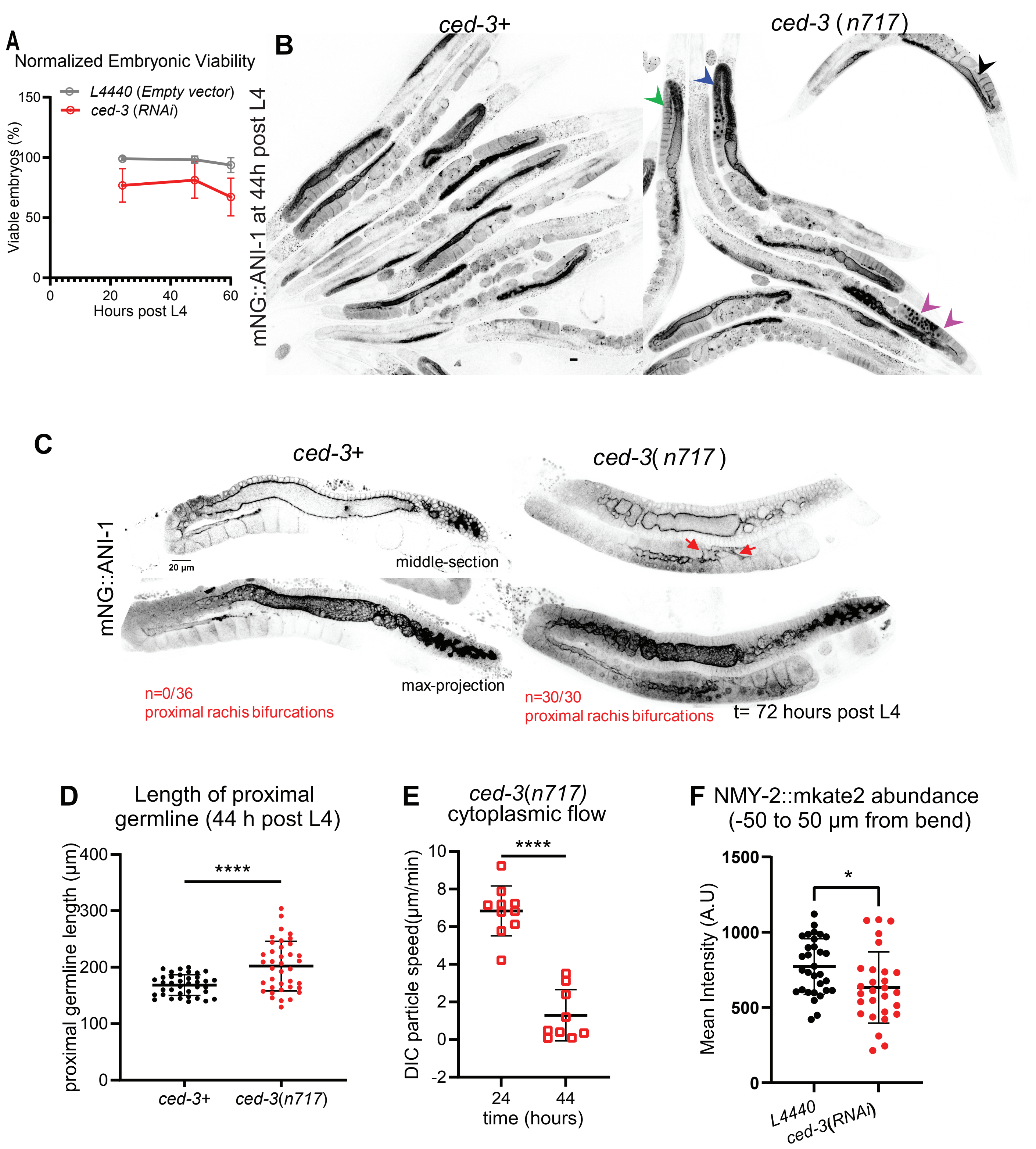

Development • Supplementary information

Fig. S1. Brood size and germline architecture defects intensify over the reproductive life of *ced-3*(*lf*) hermaphrodites.

A. Quantification of embryonic viability in the conditions indicated where L4 worms were exposed to control and RNAi conditions for 24-,44- and 60 h. Bars on graph indicate mean ±s.d., three independent experiments, *n*≥10 mothers per condition analyzed. **B.** Representative germline morphologies observed in *ced-3*(*lf*) animals at 44h post L4 are shown. Arrowheads point to normal (Black), clustered (Blue), stacked (Green), clustered germ compartment nuclei and stacked oocytes (Magenta). **C.** Representative images showing germline morphology via mNG::ANI-1, at post-fertility age (L4+72h) in the indicated genotypes, red arrows point to bifurcations of the rachis observed in *ced-3* mutant germlines. **D.** Length of the proximal germline, *n*=38 germlines per genotype, Statistical significance was determined via Welch's t test, ****p<0.0001. Error bars: mean ± s.d. **E.** Quantification of DIC particle flow speeds in the late pachytene region of *ced- 3*(*n717*) hermaphrodites at time post L4 indicated. *n*=8 animals per genotype, 1-2 particles were tracked per germline analyzed (8 total germlines were sampled for each genotype). Statistical significance was determined via Welch's t test, ****p<0.0001. Error bars: mean ± s.d. **F.** Quantification of NMY-2 intensity on rachis bridges through the germline bend for the indicated conditions, at 44h post L4. For each condition, *n*=6 animals. Statistical significance was determined by a 2-tailed student T-Test, *p= 0.0150. Error bars: mean± s.d.

Development • Supplementary information

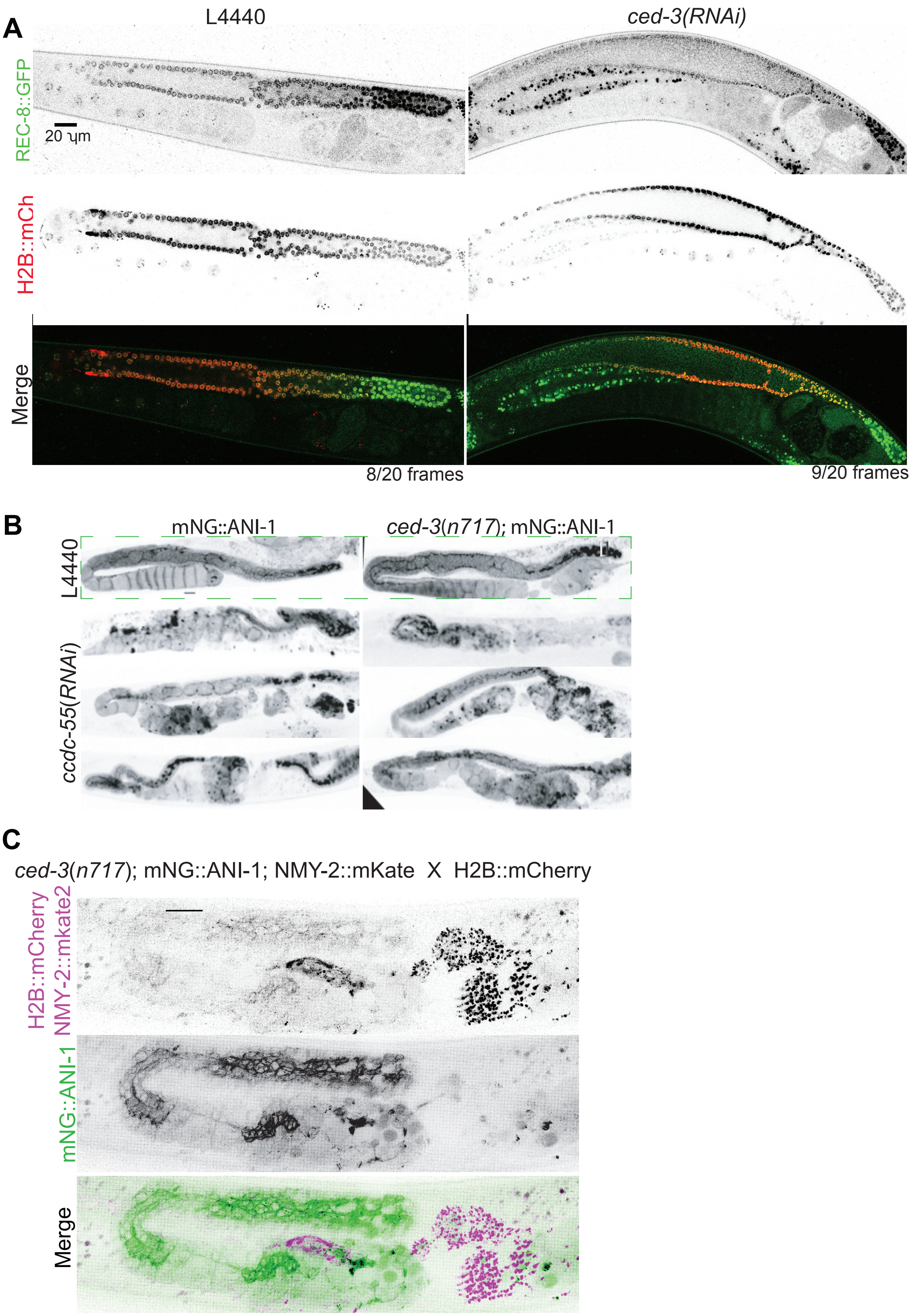
A
L4440
ced-3(RNAi)
REC-8::GFP
20 μm
H2B::mCh
Merge
8/20 frames
9/20 frames
B
mNG::ANI-1
ced-3(n717); mNG::ANI-1
L4440
ccdc-55(RNAi)
C
ced-3(n717); mNG::ANI-1; NMY-2::mKate X H2B::mCherry
H2B::mCherry
NMY-2::mkate2
mNG::ANI-1
Merge

Fig. S2. Perturbing germline shape or provision of sperm from control males does not rescue germline architectural defects of *ced-3(lf)* hermaphrodites. A. Representative germline images for all the conditions indicated (middle frame displayed) showing germline nuclei with H2B::mCherry and REC-8::GFP, *n*=3 replicated experiments. B. Z-projections of representative images showing that altering distal germline morphology via *ccdc-55(RNAi)* in the indicated genotypes failed to rescue germline architectural defects, *n*=3 independent experiments. RNAi feeding was performed for 44 hours post L4 stage for all conditions. C. Image panel shows a representative germline of *ced-3(lf)* hermaphrodites where sperm was introduced from normal males expressing H2B::mCherry marking sperm DNA, *n*=3 replicate experiments. Scale Bars: 20 μm.

Table S1. Strains used

Strain name	Genotype	Source
N2	Wild-type Bristol strain	
MT1522	*ced-3(n717) IV*	*(Ellis and Horvitz, 1986)*
MDX29	*ani-1(mon7[mNeonGreen^3xFlag::ani-1]) III*	*(Rehain-Bell et al., 2017)*
MDX40	*nmy-2(cp52[nmy-2::mkate2 + LoxP unc119(+) LoxP]) I; ani1(mon7[mNeonGreen^3xFlag::ani-1]) III;; unc-119 (ed3) III*	*(Rehain-Bell et al., 2017)*
MDX129	*nmy-2(cp52[nmy-2::mkate2 + LoxP unc119(+) LoxP]) I; ani1(mon7[mNeonGreen^3xFlag::ani-1]) III;; unc-119 (ed3) III ; ced-3(n717) IV*	*This study*
MDX155	*ltIs81 [Ppie-1::gfp-TEV-Stag::ani-2; unc119 (+)]; mex-5p::H2B::mCherry II; unc119(ed3) III*	*This study*
MDX187	*ltIs81 [Ppie-1::gfp-TEV-Stag::ani-2; unc119 (+)]; mex-5p::H2B::mCherry II; unc119(ed3) III ;ced-3 (n717) IV*	*This study*
UM208	*unc-119(ed3) III; ltIs81 [Ppie-1::gfp-TEV-Stag::ani-2; unc-119 (+)]; ltIs44 [Ppie1::mCherry::PH(PLC1delta1); unc-119(+)] IV*	*(Amini et al., 2014)*
MDX63	*ani-1(mon7[mNeonGreen^3xFlag::ani-1]) III;; ltIs44 [Ppie1::mCherry::PH(PLC1delta1); unc-119(+)] IV*	*This study*
KLG049	*naSi2[mex-5p::H2B::mCherry::nos-2 3'UTR] II*	*(Roy et al., 2018)*

Development • Supplementary information

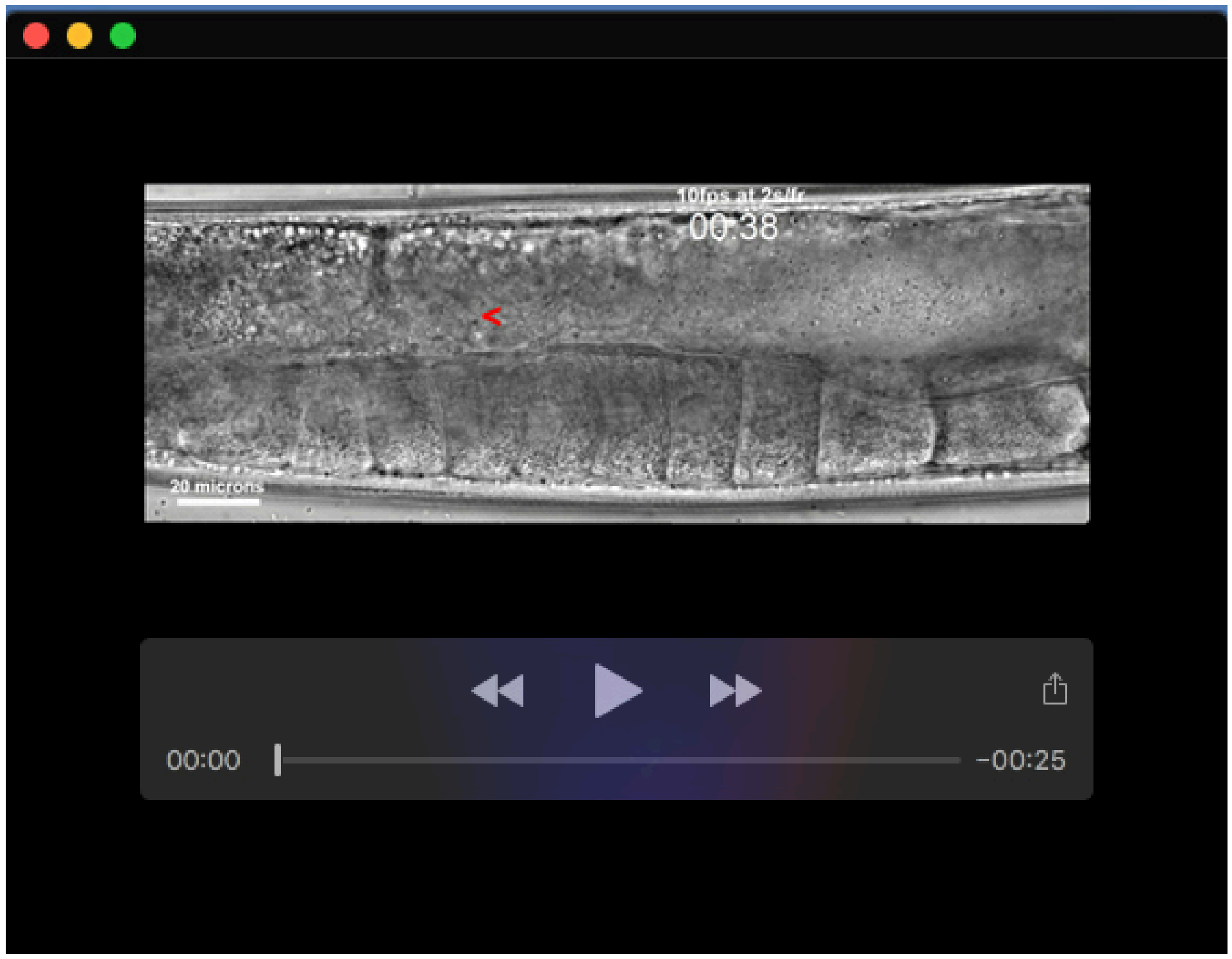

Movie 1. Cytoplasmic flows in N2, controls.

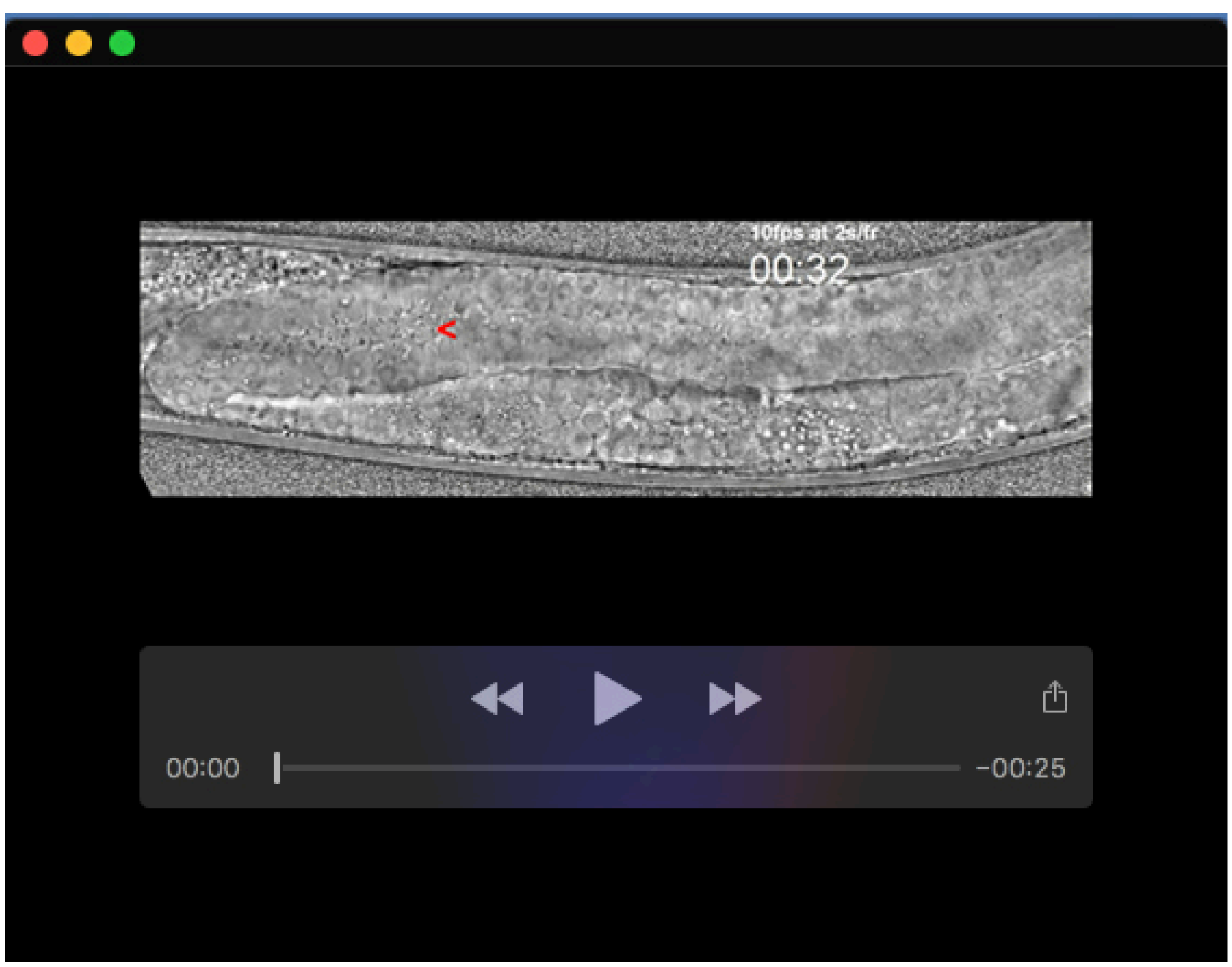

Movie 2. Cytoplasmic flows in *ced-3*(*lf*) animals (MT1522) at 44 hours post L4.

Development • Supplementary information
