# Supplementary material for: Apoptosis promotes fertility in Caenorhabditis elegans by maintaining functional germline morphology
Source: Development. 2026 Aug 13;153(15):dev205442. doi: 10.1242/dev.205442 (PMC13535102; doi:10.1242/dev.205442)
Supplement: Supplementary file 2 [file develop-153-205442_review_history.pdf]

Apoptosis promotes fertility in *Caenorhabditis elegans* by maintaining functional germline morphology

Udodirim N. Saydee-Onwubiko, Michael E. Werner, Gavin C. Trapp and Amy S. Maddox
DOI: 10.1242/dev.205442

Editor: Swathi Arur

Review timeline

Original submission:	11 December 2025
Editorial decision:	29 December 2025
First revision received:	3 June 2026
Editorial decision:	22 June 2026
Second revision received:	10 July 2026
Accepted:	13 July 2026

Original submission

First decision letter

MS ID#: dev.205442

MS TITLE: Apoptosis promotes fertility in C. elegans by maintaining functional germline morphology

AUTHORS: Udodirim Saydee-Onwubiko; Michael E. Werner; Gavin C. Trapp; Amy Shaub Maddox

Dear Amy,

I have now received all the referees' reports on the above manuscript, and have reached a decision. The referees' comments are appended below.

As you will see, the referees express considerable interest in your work, and also provide significant recommendations both experimental and textual to further test the models in the study for additional rigor and some underlying mechanism for the phenomenon observed. If you are able to revise the manuscript along the lines suggested, which may involve further experiments, I will be happy to receive a revised version of the manuscript. Your revised paper will be re-reviewed by one or more of the original referees.

Please attend to all of the reviewers' comments and ensure that you upload both a 'clean' version of your Word file, along with a highlighted version clearly showing where you have made changes in the revised manuscript. Please avoid using 'Tracked changes' in Word files as these are lost in PDF conversion. I should be grateful if you would also provide a point-by-point response detailing how you have dealt with the points raised by the reviewers in the 'Response to Reviewers' box. If you do not agree with any of their criticisms or suggestions please explain clearly why this is so.

Reviewer 1

SUMMARY OF THE ADVANCE MADE IN THIS PAPER AND ITS POTENTIAL SIGNIFICANCE TO THE FIELD

© 2026. Published by The Company of Biologists under the terms of the Creative Commons Attribution License (https://creativecommons.org/licenses/by/4.0/).

The manuscript by Saydee-Onwubiko et al. reports on the phenotypic characterization of the C. elegans germline in mutants that cannot undergo apoptosis. They show that ced-3(lf) mutants have progressive germline architecture defects as animals progress through adulthood, with changes in rachis diameter, germ cell organization and oocyte size. They further report that meiotic progression is slowed, particularly in the mitosis-to-meiosis transition zone, and that the smaller oocytes likely impact embryonic viability. They propose that the lack of apoptosis is initially compensated by gonad expansion and cell cycle delays until the accumulation of germ cell compartments eventually results in compromised germline architecture and oocyte growth, impacting embryonic viability.

Overall I find the manuscript to be of excellent quality: the experimental design is sound and the findings that are reported are novel. The authors also settle a discrepancy in the literature regarding the impact of apoptotic defects in C. elegans fertility and embryonic viability.

SUGGESTIONS TO AUTHORS

My main criticism is that the work remains very descriptive, and the authors do not directly seek to test their model that the increase in the number of germ cell compartments resulting from the absence of apoptosis leads to germline architecture defects and reduced oocyte size, compromising embryonic viability. The mechanistic basis for the observed defects therefore remains unclear. One possible way to test this model could be to assess whether the observed phenotypes are suppressed, even partially, in conditions that alleviate germ cell packing. This could perhaps be achieved in conditions in which ovulation is accelerated, for instance by conducting analyses in goa-1 mutants, in which ovulation occurs independently of MSP signaling (PMID: 16824915) or after treatment with serotonin, which provokes ovulation (PMID: 9130699). It could also perhaps be achieved by conducting analyses in lon-2(-) animals, which are larger than wild-type and thus could delay the appearance of germline architecture defects, or in ccdc-55-depleted animals, which have an extended distal gonad and thus could likely accommodate more germ cell compartments (PMID: 22285439).

Related to the mechanistic basis of the observations, I find it surprising that the authors did not measure cytoplasmic streaming in the rachis of ced-3(lf) mutants. The authors correctly point out that streaming was reported to be normal by Wolke et al (2007), yet it is unclear when in adulthood measurements were conducted in the context of this latter study. Have the authors considered the possibility that streaming in ced-3(lf) mutants is minimally perturbed during the first 24h of adulthood but becomes progressively compromised as animals age and defects are more prominent? The authors find that the diameter of the rachis is increased in older ced-3(lf) mutants, and I find it unlikely that streaming would not be impacted by such change in rachis volume. Decreased streaming in older ced-3(lf) animals would be compatible with a model in which oocyte expansion progressively becomes physically limited by the increase in the number of germ cell compartments. I think that it would be useful to revisit whether cytoplasmic streaming becomes progressively compromised in the rachis of ced-3(lf) animals.

Minor suggestions:

The authors conclude that ced-3(lf) animals display cell cycle delays that partly compensate for the extra germ cell compartments. The conclusion is mainly based on the visual scoring of nuclei that reveals an increase in the size of the transition zone. I somewhat find this analysis limiting, as the images provided make it difficult for non-experts to come to the same conclusion (the quality of the images did not enable me to see this). I think that this conclusion would be significantly strengthened if the authors had an independent mean to demonstrate the increase in the size of the transition zone (perhaps, for instance, by scoring him-3 and rec-8). Scoring rec-8 would have the added benefit of more directly assessing whether germ cells have defects in meiotic entry (which, as I understand, is somewhat implied by the authors' statement that there is a "delayed exit from the transition zone in ced-3(lf) animals").

At the end of the first result section, the authors conclude that "morphological anomalies develop before exhaustion of the sperm supply and intensify as animals age". Is it clear that sperm is not depleted at this stage in ced-3(lf) animals? If the authors have data to show this, or if this has been assessed by another study, it would be useful to report it here.

© 2026. Published by The Company of Biologists under the terms of the Creative Commons Attribution License (https://creativecommons.org/licenses/by/4.0/).

Most of the assays done by the authors are conducted on ced-3(lf) homozygotes, in which no cell death will occur, including in the soma. Some aspects of apoptosis were previously shown to be controlled non-cell autonomously (e.g. PMID: 20137949). Could some of the observed germline phenotypes result from non-cell autonomous defects arising of the extra somatic cells present in ced-3(lf) animals? The authors could consider assessing this by conducting ced-3 depletion only in the germline (for instance using a germline-expressed rde-1 strain). Finding that the phenotype is autonomous to the germline would help to strengthen the authors' proposed model.

p.8, 3rd paragraph: The mammalian mpk-1 homologs are ERK1 and ERK2.
P.9, 2nd paragraph: The fact that loss OF apoptosis also…

Reviewer 2

Saydee-Onwubiko et al. report a role for apoptosis (programmed cell death) in oogenesis using C. elegans as a model organism. Previous studies have shown that apoptosis-defective animals are viable and fertile; however, other work has demonstrated that loss of apoptosis leads to germline hyperplasia and reduced oocyte quality in aged animals. In this study, the authors perform a detailed anatomical analysis of germline architecture in apoptosis-defective animals during reproduction. The apoptosis-defective germline exhibits several morphological defects, including abnormal rachis organization and reduced size of germline compartments approximately 40 hours after the fourth larval (L4) stage, resulting in the production of smaller oocytes. Notably, loss of apoptosis also causes a delay in exit from the transition zone, where germ cells undergo the mitosis-to-meiosis switch.

Overall, the experiments are well designed, and most results are supported by appropriate quantitative analyses. Although this manuscript is largely descriptive in its characterization of germline morphology, the discovery that meiotic cell-cycle progression and rachis architecture are coordinated with cell death provides new insight into the role of apoptosis in animal reproduction. Based on these considerations, I am generally supportive of its publication in Development, provided that the authors address the following minor points:

1) To clarify whether the abnormal oocyte shapes arise from defects in maternal and/or paternal factors, the authors may want to examine germline architecture and mNG::ANI-1 distribution in ced-3(n717) hermaphrodites mated with wild-type males.

2) The authors should clearly state in the Methods section whether RNAi against ced-3 was performed for a uniform 24-hour period in all experiments, or whether treatments were maintained for 24, 44, or 60 hours from the L4 stage.

3) It is somewhat difficult to understand how delayed exit from the transition zone could compensate for morphological defects during and after the pachytene stage. Additional discussion of the potential molecular mechanisms underlying this proposed compensation would be appreciated.

Reviewer 3

Summary:

Saydee-Onwubiko et. al, employ loss-of-function of the germline apoptosis caspase CED-3 in *C. elegans* to probe how apoptosis promotes fertility and oocyte quality during oogenesis. They report that nematodes lacking apoptosis display fertility defects (brood size reduction) that increase in severity with age, and age-dependent increase in defects in germline architecture. Defects increase in germline compartment number, decrease in germ cell size, and increase in area of the net cytoplasm donor rachis cross section. They also postulate that fertilization of smaller oocytes that fail to develop results in the observed age-dependent embryonic lethality. They report that these architectural and fertility defects are in part compensated by delayed cellularization.

© 2026. Published by The Company of Biologists under the terms of the Creative Commons Attribution License (https://creativecommons.org/licenses/by/4.0/).

Major concerns-

This is an interesting study that looks to resolve prior reports pertaining to the poorly understood problem of oocyte quality. While the analyses presented are thorough, the study offers no molecular insight and is highly descriptive, resting on various correlations rather than direct causations. Providing molecular mechanism would be important. For example, a very feasible addition would be to experimentally expand on the ERK-1/MPK-1 point mentioned in the Discussion (line numbers were not provided).

Minor concerns-

1. Figure 1D and E should include arrows pointing to key structural differences.
2. Why is the *ced-3(n717)* allele used most of the time, but *ced-3* RNAi other times (Figure 2A, 3F)?
3. Why are statistics not represented in Figure 3B (though described in the figure legend)?

Suggestion for better mechanistic understanding:

Could the authors try examining the a *ced-3* hypomorph? The *ced-3(n2427)* allele is a temperature-sensitive hypomorphic allele. It would be interesting to see of an intermediate level of the above defects and compensatory responses are seen.

Apoptotic markers-though known zone

Other apoptotic genes egl-1?

Expression and localization of CED-3?

Timing—heat shock of C ED-3 action

First revision

Author response to reviewers' comments

We are grateful for the review of our manuscript and thank the reviewers for their insights on ways to improve our manuscript. We also appreciate the editorial staff's permission for extended time to complete revisions as one of us devoted important time to early parenthood. We have now addressed all the reviewers' concerns:

Please note that that line numbers referenced here refers to the marked-up version of the manuscript.

Reviewer concerns (black font), Response to concerns (blue font)

The manuscript by Saydee-Onwubiko et al. reports on the phenotypic characterization of the C. elegans germline in mutants that cannot undergo apoptosis. They show that ced-3(lf) mutants have progressive germline architecture defects as animals progress through adulthood, with changes in rachis diameter, germ cell organization and oocyte size. They further report that meiotic progression is slowed, particularly in the mitosis-to-meiosis transition zone, and that the smaller oocytes likely impact embryonic viability. They propose that the lack of apoptosis is initially compensated by gonad expansion and cell cycle delays until the accumulation of germ cell compartments eventually results in compromised germline architecture and oocyte growth, impacting embryonic viability.

© 2026. Published by The Company of Biologists under the terms of the Creative Commons Attribution License (https://creativecommons.org/licenses/by/4.0/).

Overall I find the manuscript to be of excellent quality: the experimental design is sound and the findings that are reported are novel. The authors also settle a discrepancy in the literature regarding the impact of apoptotic defects in C. elegans fertility and embryonic viability.

We are gratified that the reviewer appreciated our experimental design and the advance that our novel findings made possible.

SUGGESTIONS TO AUTHORS

My main criticism is that the work remains very descriptive, and the authors do not directly seek to test their model that the increase in the number of germ cell compartments resulting from the absence of apoptosis leads to germline architecture defects and reduced oocyte size, compromising embryonic viability. The mechanistic basis for the observed defects therefore remains unclear. One possible way to test this model could be to assess whether the observed phenotypes are suppressed, even partially, in conditions that alleviate germ cell packing. This could perhaps be achieved in conditions in which ovulation is accelerated, for instance by conducting analyses in goa-1 mutantos, in which ovulation occurs independently of MSP signaling (PMID: 16824915) or after treatment with serotonin, which provokes ovulation (PMID: 9130699). It could also perhaps be achieved by conducting analyses in lon-2(-) animals, which are larger than wild-type and thus could delay the appearance of germline architecture defects, or in ccdc-55-depleted animals, which have an extended distal gonad and thus could likely accommodate more germ cell compartments (PMID: 22285439).

We thank the reviewer for their interest in testing whether the observed architectural defects of *ced*(*lf*) animals' germlines could be alleviated/rescued. Since some of the other proposed mutants are known to have other oogenic defects, we decided to target CCDC-55 to test whether elongating the germline could, by accommodating the longer cell cycle zones within the distal region of the germline in *ced-3*(*lf*) animals, rescue the oogenic defects in the proximal germline. We first subjected *ced-3*(*lf*) animals to *ccdc-55(RNAi)* starting in the first larval stage, and examined their germlines 44 hours after L4. Unfortunately, CCDC-55 depletion grossly perturbed the germline in our negative controls, changing not only distal germline dimensions but also morphology (Please see Figure A below for details). To avoid perturbing oogenesis and specifically perturb (and extend) germline elongation, we repeated the experiment, stopping RNAi feeding at the fourth larval stage when germline elongation should be complete and when oogenesis commences, moving worms to normal feeding plates, and observing the phenotypes at 44 hours later. Still, depletion of CCDC-55 caused abnormal stacking and clustering of oocytes, and other abnormalities like tortuous germlines, even in our negative controls. We thus could not conclude that extending the distal germline could alleviate *ced-3*(*lf*)'s germline architectural defects. Furthermore, morphological defects persisted in *ced-3*(*lf*) germlines depleted of CCDC-55 and thus longer.

We have included a sentence to address these findings briefly (Please see lines 225-227 in the text), "Extending…. morphological defects (data not shown)".

NOTE: We have removed unpublished data that had been provided for the referees in confidence.

Related to the mechanistic basis of the observations, I find it surprising that the authors did not measure cytoplasmic streaming in the rachis of ced-3(lf) mutants. The authors correctly point out that streaming was reported to be normal by Wolke et al (2007), yet it is unclear when in adulthood measurements were conducted in the context of this latter study. Have the authors considered the possibility that streaming in ced-3(lf) mutants is minimally perturbed during the first 24h of adulthood but becomes progressively compromised as animals age and defects are more prominent? The authors find that the diameter of the rachis is increased in older ced-3(lf) mutants, and I find it unlikely that streaming would not be impacted by such change in rachis volume. Decreased streaming in older *ced-3*(*lf*) animals would be compatible with a model in which oocyte expansion progressively becomes physically limited by the increase in the number of germ cell compartments. I think that it would be useful to revisit whether cytoplasmic streaming becomes progressively compromised in the rachis of ced-3(lf) animals.

© 2026. Published by The Company of Biologists under the terms of the Creative Commons Attribution License (https://creativecommons.org/licenses/by/4.0/).

The reviewer makes a great point that indeed it is only later, 44 hours post L4, that *ced-3*(*lf*) animals exhibit germline architecture defects, so Wolke's report of normal cytoplasmic flow speeds in early adults does not inform our understanding of mid-adult oogenesis defects. Grateful for the reviewer's curiosity, we quantified cytoplasmic flows. The rates of flow in the late pachytene and bend regions for control and *ced-3(lf)* animals' germlines were indistinguishable at the younger adult stage (24 hours post L4 as examined by Wolke et al. 2007) (both ~7 +/- 1.5 microns/min). By contrast, 44 hours after L4, when we observed germline morphology defects, the rate of cytoplasmic flow was significantly lower in both regions in *ced-3*(*lf*) animals compared to controls. These findings have now been incorporated into Figure 3G, and S1D and the corresponding text (pg. 6, beginning at line 232 "Cytoplasmic flow speed…").

Minor suggestions:

The authors conclude that ced-3(lf) animals display cell cycle delays that partly compensate for the extra germ cell compartments. The conclusion is mainly based on the visual scoring of nuclei that reveals an increase in the size of the transition zone. I somewhat find this analysis limiting, as the images provided make it difficult for non-experts to come to the same conclusion (the quality of the images did not enable me to see this). I think that this conclusion would besignificantly strengthened if the authors had an independent mean to demonstrate the increase in the size of the transition zone (perhaps, for instance, by scoring him-3 and rec-8). Scoring rec-8 would have the added benefit of more directly assessing whether germ cells have defects in meiotic entry (which, as I understand, is somewhat implied by the authors' statement that there is a "delayed exit from the transition zone in ced-3(lf) animals").

Inspired by this suggestion, we now use a REC-8 reporter as a complementary approach to discern mitotic, transition, and pachytene nuclei. To conduct our assessments as performed previously, we first attempted crossing REC-8::GFP worms to *ced-3*(*n717*) hermaphrodites but all efforts were unsuccessful. Since we have observed similar phenotypes between loss of function allele (*ced-3*(*n717*), and CED-3 RNAi, we depleted CED-3 from animals expressing REC-8::GFP; H2B::mCh and imaged the worms at 44h post L4. Compatible with our previous findings, our analysis suggested that the transition zone visualized in this distinct genetic background was longer in CED-3 depleted animals than in controls. We have included a panel below showing our observations (Please see B, below).

Interestingly, compared to L4440 treated controls, the mitotic zone of CED-3 depleted germlines was now significantly shorter. We think this is due to a difference in RNAi strength vs the allelic disruption of CED-3 function. We have now extensively rewritten the results section to reflect our conclusions from these observations. Please see changes on beginning on Line 177 "Loss of apoptosis …. progression". We also discuss our observations (line 199-209).

NOTE: We have removed unpublished data that had been provided for the referees in confidence.

At the end of the first result section, the authors conclude that "morphological anomalies develop before exhaustion of the sperm supply and intensify as animals age". Is it clear that sperm is not depleted at this stage in ced-3(lf) animals? If the authors have data to show this, or if this has been assessed by another study, it would be useful to report it here.

We agree that including this information will improve our manuscript. We now report the presence of sperm in the spermatheca of hermaphrodites 44 hours after L4 (Red arrows, Figure C, C' below), and even at L4+60h. Thus, we are confident that the germline morphological defects we report are not due to sperm depletion. This finding has been added to (Fig. 2A) and the corresponding text (line 153-155) in our revision and shown in Figure C below.

NOTE: We have removed unpublished data that had been provided for the referees in confidence.

Most of the assays done by the authors are conducted on ced-3(lf) homozygotes, in which no cell death will occur, including in the soma. Some aspects of apoptosis were previously shown to be

© 2026. Published by The Company of Biologists under the terms of the Creative Commons Attribution License (https://creativecommons.org/licenses/by/4.0/).

controlled non-cell autonomously (e.g. PMID: 20137949). Could some of the observed germline phenotypes result from non-cell autonomous defects arising of the extra somatic cells present in ced-3(lf) animals? The authors could consider assessing this by conducting ced-3 depletion only in the germline (for instance using a germline-expressed rde-1 strain). Finding that the phenotype is autonomous to the germline would help to strengthen the authors' proposed model.

To complement our use of the *ced-3(lf)* allele, we performed CED-3 depletion, during which pre-existing CED-3 protein is flushed out via oogenesis (whereas loss of target protein in somatic cells relies on protein run-down according to a target's intrinsic half-life). Furthermore, many somatic *C. elegans* cells including neurons and some epithelial cells are highly inefficient at RNAi. For all these reasons, we expect that CED-3 depletion is most robust in the germline. *ced-3(RNAi)* and *ced-3(lf)* had essentially the same effects on germline architecture (Please see Figures 2B, 3A, F, S1A).

KRI-1 in somatic tissues promotes germ cell death in response to DNA damage (Ito, Derry, et al Curr Biol 2010). While *ced-3* ultimately regulates cell death, neither the relationship between *ced-3* and *kri-1*, nor the specific somatic tissue(s) in which KRI-1 acts, is known.
Thus, while this is an interesting avenue worth exploring, we believe it is outside the scope of this manuscript. We have now included a paragraph to acknowledge the presence of this knowledge gap in our discussion (Lines 359-374).

**-p.8, 3rd paragraph: The mammalian mpk-1 homologs are ERK1 and ERK2.
**-P.9, 2nd paragraph: The fact that loss OF apoptosis also…

We are grateful for the in-depth reading and suggestions. These sentences have now been corrected within the manuscript (Changes reflected at lines 352 ("ERK1/2..", and 384 ("of").

Reviewer 2

"Saydee-Onwubiko et al. report a role for apoptosis (programmed cell death) in oogenesis using C. elegans as a model organism. Previous studies … I am generally supportive of its publication in Development, provided that the authors address the following minor points:"

We thank the reviewer for their affirming and thoughtful suggestions.

1)To clarify whether the abnormal oocyte shapes arise from defects in maternal and/or paternal factors, the authors may want to examine germline architecture and mNG::ANI-1 distribution in ced-3(n717) hermaphrodites mated with wild-type males.

We thank reviewer 2 for this suggestion. We had not anticipated that the germline morphology/architecture defects of *ced-3*(*lf*) germlines would be associated with sperm since apoptotic removal of pachytene compartments only occurs during oogenesis (Gumienny et al.,1999).

We still wanted to address this reasonable concern. To this end, we crossed *ced-3*(*lf*) hermaphrodite adults (30 hours post L4) to control males expressing H2B::mCh and assessed germline morphology 14 hours later (at 44 hours post L4).

We did not observe any changes in germline morphology, or any rescue. Compartments were still stacked, clustered, and abnormally shaped in the mated *ced-3*(*lf*) germlines, similar to the unmated *ced-3*(*lf*) germlines (Figure D, below). These results support the idea that loss of germline apoptosis affects oogenesis by perturbing the oogenic germline itself and not via an effect on sperm quality or quantity that can be rescued by the presence of control sperm. We have included this representative image in the supplement (Figure S2).

NOTE: We have removed unpublished data that had been provided for the referees in confidence.

© 2026. Published by The Company of Biologists under the terms of the Creative Commons Attribution License (https://creativecommons.org/licenses/by/4.0/).

2) The authors should clearly state in the Methods section whether RNAi against ced-3 was performed for a uniform 24-hour period in all experiments, or whether treatments were maintained for 24, 44, or 60 hours from the L4 stage. ****

We have revised the manuscript to explain that CED-3 RNAi treatments were maintained for 24, 44, or 60 hours from the L4 stage, in each case, from L4 until the stage at which the animal was examined. The following text was included in the methods section "RNAi treatments were continuously …, and 60-hour treatments", (lines 439-442).

3) It is somewhat difficult to understand how delayed exit from the transition zone could compensate for morphological defects during and after the pachytene stage. Additional discussion of the potential molecular mechanisms underlying this proposed compensation would be appreciated.

We thank reviewer 2 for the opportunity to further explain our findings. In fact, in revising our manuscript, we performed a similar analysis of germline cell cycle progression using animals expressing REC-8::GFP to clearly visualize the mitotic and transition zones. We observed that not only is the transition zone longer, pachytene and the mitotic region as well. Since *ced-3*(*lf*) germlines are longer than controls in general we interpret these results as a shift in the normal arrangement of germ cells. The data suggests that an excess amount of germ cells can ultimately disrupt the normal arrangement of germ cells and is likely what occurs in *ced-3*(lf) germlines. We have now added an extra line in the discussion section to elaborate on how we think such shifts in the cell cycle zones could disrupt germline architecture (line 338-342).

Reviewer 3

Major concerns- This is an interesting study that looks to resolve prior reports pertaining to the poorly understood problem of oocyte quality. While the analyses presented are thorough, the study offers no molecular insight and is highly descriptive, resting on various correlations rather than direct causations. Providing molecular mechanism would be important. For example, a very feasible addition would be to experimentally expand on the ERK-1/MPK-1 point mentioned in the Discussion (line numbers were not provided).

From our pilot studies and current literature, we understand that MAPK acts differently in the soma versus the germline. Specifically, the A and B forms of MAPK that are reciprocally enriched in the soma and germline, respectively, function differently. Our next steps would be to first determine the localization of active MAPK in *ced-3(lf)* germlines, to define whether elimination of apoptosis shifts where MAPK is active. Because we foresee that the answer to this exploration will be the beginning of a new mechanistic hypothesis (line 405-406) and not a closure of our present story, we opted not to perform this work to revise our present manuscript. Our focus on the mechanical and morphological impacts of apoptosis on oogenesis has been strengthened by addition of our findings that apoptosis is required for the cytoplasmic flows that enlarge oogenic compartments.

Please see that we have now provided line numbers in our manuscript, thank you for this helpful suggestion.

Minor concerns

1: Figure 1D and E should include arrows pointing to key structural differences.

We thank our reviewer for this suggestion and provide some markings to Figure 1D. We represented structures of the germline in the schematic Figure 1C. Following Figure 1D shows the rachis as outlined in the schematic figure (1C) but in 24h post L4 germlines. Figure 1E is color coded to represent the phenotypes observed and also corresponds with the colors represented on the graph of this occurrence of these phenotypes in the population (Figure 1F). We have included some

© 2026. Published by The Company of Biologists under the terms of the Creative Commons Attribution License (https://creativecommons.org/licenses/by/4.0/).

brackets to highlight these structures in Figure 1D, and a line of text to clarify this better (Line 136).

2: Why is the ced-3(n717) allele used most of the time, but ced-3 RNAi other times (Figure 2A, 3F)?

CED-3 function has been removed via RNAi in some of the foundational papers in this field, including Andux and Ellis, PLoS Genetics 2008, Chartier, Grill et al., Nature Physics 2021. Earlier papers, including Gumienny, Hengartner et al., Development 1999, Wolke, Priess et al., Development 2007, and Das, Arur., et al., Sci Advances 2020 papers use the ced-3(*n717*) allele. For the first time that we are aware of, we combine the use of these tools and report their similarity and complementarity. Practically speaking, use of RNAi was strategic in some cases where combining the allele with certain fluorescent markers was difficult.

3: Why are statistics not represented in Figure 3B (though described in the figure legend)?

We apologize for not having presented the results of our statistical tests. The original graph has been replaced, now in supplement 1D with the appropriate statistics.

Suggestion for better mechanistic understanding:

Could the authors try examining the a ced-3 hypomorph? The ced-3(n2427) allele is a temperature-sensitive hypomorphic allele. It would be interesting to see of an intermediate level of the above defects and compensatory responses are seen.

We thank reviewer #3 for their interest in getting a better mechanistic understanding of our findings. We were unaware that *ced-3(n2427)* is thermosensitive and have not been able to find information to that effect online but would be pleased to learn more if *Development* would condone our scientific dialogue following completion of our blinded interaction in the review process. In order to tune the number of apoptotic oogenic compartments, we do aim to deplete the apoptosis inhibitor CED-9 and assess the effects on oogenesis of removing more compartments via germline apoptosis. Those studies have only just been initiated but preliminary results suggest that there is not an inverse phenotype to loss of CED-3, so unfortunately there is nothing tidy to add to this revision.

Apoptotic markers-though known zone Other apoptotic genes egl-1?

EGL-1 is indeed interesting, but it specifically regulates somatic cell apoptosis (Conradt and Horvitz, Cell 1998) and not germline apoptosis (also established by Gumienny, Hengartner et al, 1999). Since our study focuses on the oogenic effects of the absence of apoptosis, and since the effectiveness of loss of CED-3 has been established (and confirmed by us), we did not explore alternative markers for the apoptotic zone in our revisions.

Expression and localization of CED-3?

CED-3 localizes to the nuclear periphery (Chen et al., Nat Struct Mol Biol 2016), which is perhaps how it is presumably retained within dying compartments of the syncytial germline. As mentioned above, since our study focuses on the oogenic effects of the absence of apoptosis, we were less focused on the mechanisms of compartmentalizing apoptotic biochemistry when it does occur, though we do agree that that is very interesting.

Timing—heat shock of C ED-3 action

We are unaware of a thermosensitive allele of *ced-3* but agree that that would be a powerful tool.

© 2026. Published by The Company of Biologists under the terms of the Creative Commons Attribution License (https://creativecommons.org/licenses/by/4.0/).

Measuring cytoplasmic flow using PIV labs

Thank you for that suggestion, which we happily took. Please see response above to another reviewer.

Second decision letter

MS ID#: dev.205442R1

MS TITLE: Apoptosis promotes fertility in C. elegans by maintaining functional germline morphology

AUTHORS: Udodirim Saydee-Onwubiko; Michael E. Werner; Gavin C. Trapp; Amy Shaub Maddox

Dear Dr Maddox,

I have now received all the referees reports on the above manuscript, and have reached a decision. The referees' comments are appended below.

The overall evaluation is positive and we would like to publish a revised manuscript in Development. Reviewer 1 makes suggestions to clarify the text and grammar. I ask that you address these changes for clarity, and rigor. While I do not expect to send the manuscript back to the reviewer, I will look at these myself, thus I ask that you attend to all of the reviewers' comments in your revised manuscript and detail them in your point-by-point response. If you do not agree with any of their suggestions explain clearly why this is so.

Reviewer 1

SUMMARY OF THE ADVANCE MADE IN THIS PAPER AND ITS POTENTIAL SIGNIFICANCE TO THE FIELD

The revised manuscript by Saydee-Onwubiko et al. does a good job to address most of the concerns and suggestions made by the reviewers in the previous round and therefore I think that it is improved. As brought forth by all reviewers however, while the manuscript is informative, the work remains very descriptive and lacks a mechanistic basis to support the authors' findings. The new results demonstrating that cytoplasmic streaming is impacted in older ced-3(-) mutants is a step in this direction, however there is no further mechanistic information that is contributed. Reviewers 1 and 3 had made a few suggestions to address this in the previous round and only one of these (the RNAi depletion of cdcc-55) was attempted. I understand that the results from this experiment were confounding, yet the problem of a lack of mechanistic framing remains whole. The work is therefore well done and meaningful, but descriptive.

MINOR SUGGESTIONS TO AUTHORS

Lines 83-84, "Once ovulated from the uterus and fertilized, oocytes complete meiosis and begin embryogenesis": oocytes are not ovulated from the uterus, they rather ovulate from the proximal germ line into the uterus.

Lines 214-215, "As oogenic compartments proceed through prophase of meiosis I, they gradually mature, by virtue of their sustained connection with the syncytium": I do not think that it has formally be demonstrated that there is a causal relation between oocyte maturation and syncytial connection, as such demonstrations are difficult. I understand what the authors mean and I agree that this is likely how it works but I suggest rewriting the sentence to avoid implying causality.

Lines 241-242, "The proximal part of the germline was also significantly extended proximal part of the germline of ced-3(lf) animals": Something does not work in this sentence, please verify.

© 2026. Published by The Company of Biologists under the terms of the Creative Commons Attribution License (https://creativecommons.org/licenses/by/4.0/).

Lines 364-365: Please see (and cite) PMID: 17599823 for a fairly good estimate apoptotic events in the germ line.

Lines 370-371: Please consider citing PMID: 34038716, which I think is relevant here.

Reviewer 2

The authors have satisfactorily addressed all of the concerns I raised in my previous review. The new results demonstrating a male sperm-independent contribution of ced-3 strengthen the manuscript. Although I agree with the other reviewer's comment that the study remains largely descriptive, I believe that the manuscript now presents an reasonably high-quality dataset and supports a novel conclusion in the field of C. elegans germ cell development.

Reviewer 3

SUMMARY OF THE ADVANCE MADE IN THIS PAPER AND ITS POTENTIAL SIGNIFICANCE TO THE FIELD

The authors have sufficiently made their case as to the impact of their study to the field.

SUGGESTIONS TO AUTHORS

None.

Second revision

Author response to reviewers' comments

Dear Dr. Arur and colleagues,

Thank you for your continued consideration of our paper.

Our point-by-point response is below and files have been created.

Lines 83-84, "Once ovulated from the uterus and fertilized, oocytes complete meiosis and begin embryogenesis": oocytes are not ovulated from the uterus, they rather ovulate from the proximal germ line into the uterus.
This has been edited.

Lines 214-215, "As oogenic compartments proceed through prophase of meiosis I, they gradually mature, by virtue of their sustained connection with the syncytium": I do not think that it has formally be demonstrated that there is a causal relation between oocyte maturation and syncytial connection, as such demonstrations are difficult. I understand what the authors mean and I agree that this is likely how it works but I suggest rewriting the sentence to avoid implying causality.
This has been edited.

Lines 241-242, "The proximal part of the germline was also significantly extended proximal part of the germline of ced-3(lf) animals": Something does not work in this sentence, please verify.
This has been edited.

Lines 364-365: Please see (and cite) PMID: 17599823 for a fairly good estimate apoptotic events in the germ line.
This has been added.

Lines 370-371: Please consider citing PMID: 34038716, which I think is relevant here.

© 2026. Published by The Company of Biologists under the terms of the Creative Commons Attribution License (https://creativecommons.org/licenses/by/4.0/).

This has been added.

Thank you,
Amy, Udo et al

Third decision letter

MS ID#: dev.205442R2

MS TITLE: Apoptosis promotes fertility in C. elegans by maintaining functional germline morphology

AUTHORS: Udodirim Saydee-Onwubiko; Michael E. Werner; Gavin C. Trapp; Amy Shaub Maddox

Dear Dr Maddox,

I am happy to tell you that your manuscript has been accepted for publication in Development, pending our standard publication integrity checks.

© 2026. Published by The Company of Biologists under the terms of the Creative Commons Attribution License (https://creativecommons.org/licenses/by/4.0/).
